# Peptide-Hydrogel Nanocomposites for Anti-Cancer Drug Delivery

**DOI:** 10.3390/gels9120953

**Published:** 2023-12-04

**Authors:** Farid Hajareh Haghighi, Roya Binaymotlagh, Ilaria Fratoddi, Laura Chronopoulou, Cleofe Palocci

**Affiliations:** 1 Department of Chemistry, Sapienza University of Rome, Piazzale Aldo Moro 5, 00185 Rome, Italy; farid.hajarehhaghighi@uniroma1.it (F.H.H.); roya.binaymotlagh@uniroma1.it (R.B.); ilaria.fratoddi@uniroma1.it (I.F.); 2 Research Center for Applied Sciences to the Safeguard of Environment and Cultural Heritage (CIABC), Sapienza University of Rome, Piazzale Aldo Moro 5, 00185 Rome, Italy

**Keywords:** peptide, peptide-based hydrogels, nanoparticles, nanocomposite, cancer, drug delivery

## Abstract

Cancer is the second leading cause of death globally, but conventional anticancer drugs have side effects, mainly due to their non-specific distribution in the body in both cancerous and healthy cells. To address this relevant issue and improve the efficiency of anticancer drugs, increasing attention is being devoted to hydrogel drug-delivery systems for different kinds of cancer treatment due to their high biocompatibility and stability, low side effects, and ease of modifications. To improve the therapeutic efficiency and provide multi-functionality, different types of nanoparticles (NPs) can be incorporated within the hydrogels to form smart hydrogel nanocomposites, benefiting the advantages of both counterparts and suitable for advanced anticancer applications. Despite many papers on non-peptide hydrogel nanocomposites, there is limited knowledge about peptide-based nanocomposites, specifically in anti-cancer drug delivery. The aim of this short but comprehensive review is, therefore, to focus attention on the synergies resulting from the combination of NPs with peptide-based hydrogels. This review, which includes a survey of recent advances in this kind of material, does not aim to be an exhaustive review of hydrogel technology, but it instead highlights recent noteworthy publications and discusses novel perspectives to provide valuable insights into the promising synergic combination of peptide hydrogels and NPs for the design of novel anticancer drug delivery systems.

## 1. Introduction

Traditional chemotherapy is limited by the unselective distribution of drugs in both cancerous and healthy tissues, resulting in adverse and long-term side effects for the patients [1,2]. In recent years, there have been admirable efforts to develop nano-based drug delivery systems (DDSs) benefiting various controlled-release properties, including pH- and thermo-sensitive, photo-induced, enzyme-responsive, and target-specific properties, which result in an enhanced selectivity of the drugs towards cancer cells with lower dosages required to achieve the pharmacological effects [3,4,5,6,7,8,9,10]. To date, more than 30 types of different inorganic and organic nanoparticles (NPs) have been approved in the clinic. [11] With the COVID-19 pandemic, FDA-approved lipid nano-carriers for mRNA vaccines have shown the key role of nanomedicine in managing new global challenges [12]. In fact, these lipid nano-carriers are considered the largest fraction of clinically approved NPs parenterally administered. Inorganic NPs are the second biggest group of medical nanomaterials due to their unique electronic structures. Inorganic NPs include several important and unique materials such as porous silica [13], iron oxides [14,15,16], titania [17,18], and silver and gold NPs [19,20,21,22,23,24,25,26,27,28,29]. These have been the subject of many studies, such as anticancer and antibacterial, thanks to the success of therapies that accurately combine pharmaceuticals with NPs to achieve a synergic effect [30]. In drug delivery applications, nanocarriers allow gradual and controlled drug release, extend drug circulation time, and protect drugs against oxidation/degradation. Furthermore, nanocarriers can modify the physicochemical properties of drugs (hydrophilicity/hydrophobicity) in order to enhance their therapeutic index [6,31].

**Figure 1:**
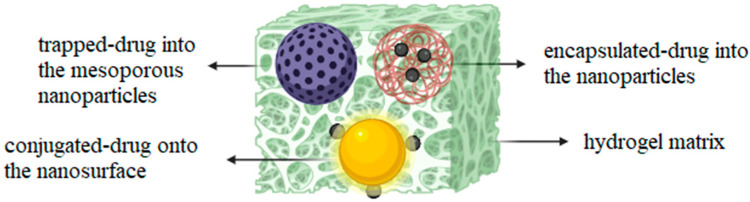
Scheme of the inclusion/incorporation of NPs in three-dimensional polymeric structures.

Hydrogels are three-dimensional (3D) polymeric networks that can adsorb and retain large amounts of water inside their matrices; they can be used as stabilizers/scaffolds for NPs to enhance their stability against aggregation and oxidation [32]. Three-dimensional hydrogel matrices can be designed in order to contain suitable functional groups for loading a variety of biomolecules and drugs. Of course, NPs may affect the mechanical and swelling features of hydrogels. [33,34,35]. Hydrogel nanocomposites are a developed class of inorganic–organic gel-type materials that may be used as drug delivery systems (DDSs), combining the benefits of both nanomaterials and hydrogels in a single platform with reinforced structures and multifunctional capabilities [36,37,38,39,40] (Figure 1). Hydrogel nanocomposites should have some basic properties for being used as DDS with maximum efficacy: (1) exhibit excellent mechanical strength and injectability to enhance in vivo drug stability as well as in situ protection/retention of drugs [41,42,43,44]; (2) have strong drug-carrier interactions to guarantee controlled and sustained drug release; (3) be structurally tunable for providing different release profiles based on the type of drug; and (4) have the capacity/ability to load several therapeutic components inside the hydrogel matrix to imply a synergic efficacy and multifunctionality.

The incorporation of NPs within hydrogels creates multifunctional systems to achieve tunable delivery systems. Besides improving the mechanical properties of the hydrogels, the presence of NPs modulates the response of the nanocomposites to different stimuli (e.g., electric, magnetic, and light-responsive hydrogels), as shown in Table 1.

Based on the source and origin of the hydrogels, the obtained nanocomposites may be classified as natural or synthetic systems. Peptide hydrogels are an interesting class of materials made from amino acid precursors. They are generally biocompatible and suitable for biological applications, including anticancer drug delivery [48,49]. Over the past decades, considerable developments in the synthesis and technology of non-peptide hydrogel nanocomposites have qualified them as promising candidates for designing controlled-release drug delivery systems. Comparatively, there is little knowledge of peptide-based hydrogel nanocomposites [50], so this review aims to highlight recent advances in the use of peptide-based nanocomposites as anticancer drug delivery vehicles. The inclusion/incorporation of nanoparticles in three-dimensional (3D) peptide hydrogels is an innovative strategy for obtaining multifunctionality, adding synergistic benefits to the new 3D structures, which are the main focus of this short but comprehensive review.

## 2. Drug Delivery Using Hydrogel Nanocomposites

### 2.1. Formulation Methods of Peptide-Hydrogel Nanocomposites

To fabricate advanced hydrogel nanocomposites and obtain the desired properties, there are four main methods, including blending, grafting, in situ precipitation, and swelling [49]. Among these methods, the swelling strategy is an in situ methodology in which the hydrogel nanocomposites are synthesized in one step. On the other hand, blending and grafting methods are considered ex situ strategies, and the nanocomposites are prepared in multiple steps [51].

In the blending method, the nanoparticles are first synthesized and then mixed with hydrogel precursors, followed by gel formation. Despite its simplicity, this method sometimes suffers from the interference of nanoparticles with gel formation, which negatively affects the final structure of the nanocomposite. Also, the lack of uniform distribution of the nanoparticles might result in their diffusion outside the hydrogel matrix upon swelling.

The grafting methods (grafting-through, grafting-from, and grafting-onto) consist of the use of grafted/grafting nanoparticles having suitable functional groups to allow the covalent bonding of nanoparticles with the peptide monomers during gel formation. Despite this advantage, the long, high-cost, and complicated fabrication process limits its use in nanocomposite preparation [51].

In the in situ precipitation method, nanoparticles are prepared in situ inside the hydrogel network during gel formation. This strategy should be performed in mild conditions (neutral pH, room temperature) and is, therefore, suitable for nanoparticles having mild condition synthesis processes. In the swelling method, after hydrogel synthesis, the nanocolloids are incubated with the gel network, which is useful for the development of microgels [51].

The intermolecular interactions among peptides in hydrogel nanocomposites are usually non-covalent (e.g., hydrogen bonds, electrostatic interactions, van der Waals, π-π stacking) and different secondary structures (α-helix, β-sheet, β-hairpin) can be formed [32].

Considering the number of hydrophobic drugs used in the clinic and those currently under development, hydrogels-DDSs for hydrophobic drugs could offer multiple advantages to patients and clinicians.

To deliver hydrophobic drugs, these could be covalently incorporated within a hydrogel network. A similar effect can be obtained using hydrophobic crosslinkers, resulting in amphiphilic hydrogels [52].

Regarding drug release from the nanocomposites, there are three main mechanisms:

1. Passively controlled drug release: Drug molecules in the hydrogel matrix are passively released through swelling and diffusion.
2. Stimuli-responsive drug delivery: Smart hydrogels drastically change their volume in response to environmental stimuli (e.g., pH, temperature, magnetic field, light, and chemical signals), triggering the release of the loaded drug.
3. Site-specific drug delivery: Drug delivery primarily relies on the properties of the hydrogel network comprising targeting components (e.g., antibodies, aptamer, folate, peptides), which allow nanoparticle design to be only focused on tuning the drug release. Such a hybrid strategy provides an opportunity to design highly specific DDSs [53].

### 2.2. Peptide-Based Hydrogel Nanocomposites Containing Inorganic NPs

As a prevalent malignancy, lung cancer has been a leading cause of cancer-related deaths in males globally [54], and non-small cell lung cancer (NSCLC) is the most common type (85% of all lung cancers [55]. For NSCLC treatment, there have been different strategies, such as targeted therapy, chemotherapy, surgical resection, radiotherapy, and immunotherapy. However, the overall survival rates of NSCLC are low. Therefore, it is vital to improve current methods [56,57], for instance, by the advancement of novel drugs and combined methods to extend them to a broader range of patients and improve overall outcomes in NSCLC.

**Figure 2:**
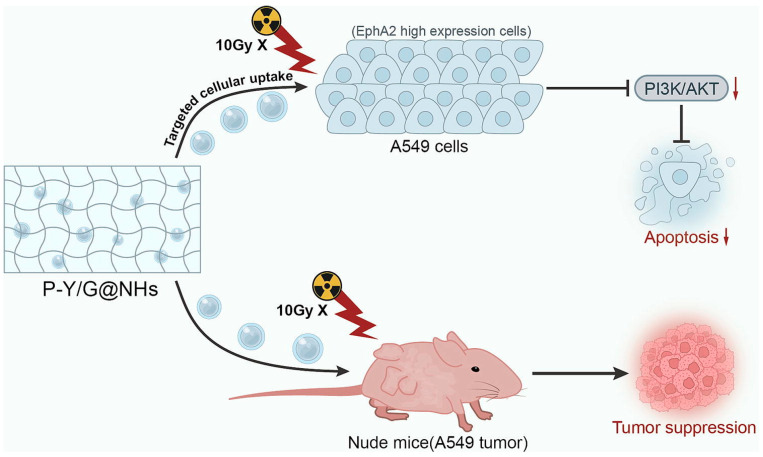
Schematic representation of in vivo and in vitro studies of YSAYPDSVPMMS (YSA) peptide-modified gold NPs-dextran-hydrogel. Reprinted with permission from [68]. Copyright 2023, Elsevier.

Paclitaxel (PTX) is a well-known clinical anticancer agent [58], widely employed as a radiosensitizer [59], capable of overcoming hypoxia-inducible factor-1α-induced radioresistance of human lung adenocarcinoma cells [60]. For PTX-targeted delivery, YSAYPDSVPMMS (YSA) peptide was successfully tested to selectively target Ephrin type-A receptor 2 (EphA2) activation when anchored with liposomal docetaxel (a common clinical formulation of PTX) in A549 lung cancer cell line [61]. Moreover, succinic anhydride (SA)-modified NPs were previously evaluated as biodegradable and biocompatible DDSs (with no anticoagulant activity) to deliver cisplatin to lung cancer cells in nude mice [62]. Regarding the inorganic radiosensitizers for radiotherapy, gold NPs are well researched in this field owing to their high X-ray absorption and unique physicochemical properties, inertness, easy synthetic method, well-known chemistry, and more importantly, their potent anticancer effects in lung cancer due to their ability to generate reactive oxygen species (ROS) in A549 cells and subsequent cell apoptosis [63]. It has been shown that a combination of such therapeutic NPs and hydrogel technology results in advanced and multifunctional nanocomposites for achieving highly effective local drug delivery [53]. In such applications, the surface of NPs should be precisely modified by suitable stabilizers. In this sense, dextran was recently employed as both a stabilizing and reducing reagent for preparing biocompatible gold NPs with high monodispersity [64]. In general, dextran-based radioconjugates can enhance the therapeutic effect of radiotherapy, and dextran uptake further accompanies the activation of the phosphatidylinositol 3-kinase/protein kinase B (PI3K/AKT) signaling pathway upon entry of viruses into cells [65,66], which can enhance the sensitivity of NSCLC radiotherapy [67]. Based on the above considerations, in 2023, Zhang et al. evaluated the effects of a nano-DDS composed of YSAYPDSVPMMS (YSA) peptide-modified gold NPs-dextran-hydrogel loaded with paclitaxel-succinic anhydride (P-Y/G@NHs) on NSCLC cell radiosensitivity. P-Y/G@NHs hydrogel nanocomposite was prepared using the following three-step method: (1) combining SA and PTX to obtain PTX-SA; (2) chemical conjugation of YSA-peptide to the PTX-SA by *N*-hydroxysuccinimide (NHS) and dicyclohexylcarbodiimide (DCC) coupling reagents to prepare the drug (PTX-SA-YSA); (3) self-assembly of polyethylene glycol-modified gold NPs, dextran, and PTX-SA-YSA to form the resultant hydrogel drug delivery system. The therapeutic properties of the P-Y/G@NHs in NSCLC cells were studied by monitoring the PI3K/AKT signaling pathway and evaluating apoptosis, colony formation, and reactive oxygen species (ROS) generation of A549 cells under 10Gy X-ray irradiation. The authors also successfully tested the in vivo therapeutic effect of this nanocomposite on A549 tumor-bearing mice. The results showed that P-Y/G@NHs can reduce the number of colonies of A549 cells by inducing both ROS production and cell apoptosis under 10Gy X-ray irradiation. In fact, the radiosensitivity of A549 cells was enhanced using P-Y/G@NHs by inhibiting the PI3K/AKT signaling pathway. The in vivo fluorescence results confirmed effective targeting and accumulating of P-Y/G@NHs at the tumor site in nude mice to enhance the radiosensitivity of target tumors without significant side effects or immune toxicity, which highlighted the potential application of P-Y/G@NHs in radiotherapy of NSCLC cells by repressing the PI3K/AKT signaling pathway (Figure 2) [68].

Other reports on peptide-based hydrogels loaded with inorganic nanoparticles for anticancer drug delivery, alongside their main advantages and disadvantages, are reported in Table 2.

### 2.3. Peptide-Based Hydrogel Nanocomposites Containing Organic NPs

One of the main drawbacks of conventional chemotherapy is the acquisition of multiple drug resistance (MDR) and systematic toxicity towards currently used small therapeutic molecules [73,74,75,76,77,78,79]. Combination cancer therapy, combining two or more drugs, enhances the treatment efficacy compared to the mono-therapy approach because it provides synergistic or additional mechanisms to damage cancer cells. This approach also potentially reduces drug resistance [80,81,82] because (1) different drugs exhibit varying pharmacokinetics and tissue distribution patterns; (2) each drug can have its own function through varying downstream/upstream mechanisms intracellularly, thus demanding drug delivery in chronological order. In fact, clinical studies on this controlled muti-drug delivery have shown an improved response rate and extended survival of patients [83,84,85], so it is highly desirable to develop DDSs that have the ability to differentially release multiple drugs.

**Figure 3:**
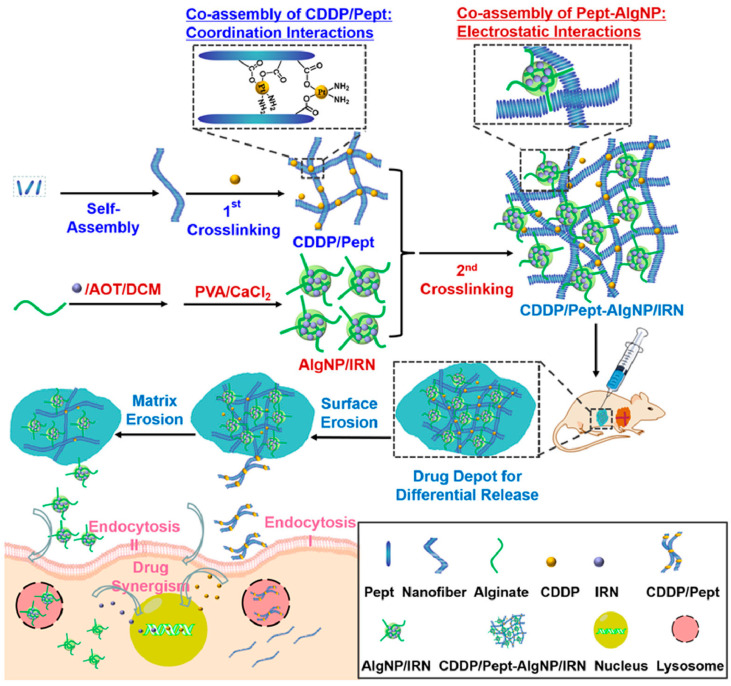
Schematic illustration of double-crosslinked cisplatin/Pept-AlgNP/irinotecan nanocomposite hydrogel for differential release of cisplatin (CDDP) and irinotecan (IRN) in combination therapy. Reprinted with permission from [98]. Copyright 2020, Elsevier.

In 2020, Wu et al. reported the synthesis of a new hydrogel nanocomposite as a DDS (abbreviated as cisplatin/Pept-AlgNP/irinotecan) composed of alginate NPs (AlgNP) and peptide-based hydrogels for delivering two clinically used anticancer drugs (irinotecan and cisplatin), which were loaded separately in the two different domains of the nanocomposite matrix. The authors specifically selected these two drugs based on previous clinical studies demonstrating a low probability of side effects with a higher response rate when irinotecan was administered after cisplatin [86] because the topoisomerase I inhibitory activity of irinotecan can be improved by cisplatin [87,88]. The designed peptide hydrogel exhibited biocompatibility and injectability thanks to its tunable amino acid sequence and peptide composition [89,90,91,92,93,94], in which the carboxyl residues of peptides form covalent bonds with cisplatin and reinforce the supramolecular self-assembly of peptide conjugates [95,96,97]. Afterward, AlgNPs were incorporated into the hydrogel matrix to act as the inner domain, to further enhance the mechanical strength of hydrogel by electrostatic interactions and significantly facilitate the encapsulation of the second drug, irinotecan, to achieve differential release profiles (Figure 3). These covalent and non-covalent interactions increased the storage capacity of the hydrogel nanocomposite up to 42-fold, compared with the native peptide gel. This drug formulation guaranteed a fast release of cisplatin before a controlled release of irinotecan, resulting in excelling synergism of the drugs in cell inhibition studies (compared to the simple mixture of the drugs), with efficacious antitumor potency confirmed by the in vivo study against A549-xenografted tumor in mouse. These studies pave the way towards the development of mechanically stable hydrogel nanocomposites as DDSs for loading individual drugs in co-assembled structures/domains to temporally control drug release and address clinical demands for combination therapy [98].

Diphenylalanine (FF) is an aromatic dipeptide extracted from Alzheimer’s *β*-amyloid polypeptide as a core recognition motif for molecular self-assembly [99]. Reches and Gazit first reported the synthesis of diphenylalanine nanotubes hydrogels (FNTs), and ever since, different advanced nanostructures, e.g., nanofibrils and spherical vesicles, have been prepared from FNTs building blocks [100], by the self-assembly of FFs via π-π interactions of the aromatic rings and backbone–backbone hydrogen bonds [101,102,103,104]. For drug delivery purposes, these biocompatible peptide-based FNTs are superior to carbon nanotubes (CNT) due to the potential toxicity of CNT in clinical studies [105].

To prepare smart DDSs, FF self-assembled structures can be conjugated with targeting ligands to become highly selective to specific cancer cells [103,106,107], with potential applications in theranostics, e.g., combined active-drug delivery with imaging tools [108]. As a well-studied targeting molecule, folic acid (FA) has a key role in the targeted delivery of various pharmaceutics because of the overexpression of folate receptors (FR) by most advanced tumors [109,110], such as brain, lung, and breast cancers. Besides folate-based drug delivery, this technology has been successfully used for other therapeutic applications, e.g., selective fluorescence, MRI, and radio imaging of cancer cells [111,112].

Among the inorganic NPs mentioned in the introduction section, magnetic NPs (MNPs) are considered a special class with high application potential in clinical studies due to their unique superparamagnetism, biocompatibility, well-known chemistry, and low cost. MNPs are being extensively investigated as the next generation of MRI contrast agents, magnetic-based DDSs, and hyperthermia agents [113,114,115,116].

FA conjugation to nanotubes and NPs has been investigated through both covalent and non-covalent strategies. Covalent bonding is superior and preferable due to its several advantages such as colloidal stability at different physiological conditions [117,118,119].

Surface coating/modification is one of the main strategies used to decrease the risks of NPs and design safer nanotechnological devices. In fact, the coating material, if chosen correctly, provides biocompatibility and affects the fate (e.g., accumulation, degradation, excretion) and the behavior (e.g., colloidal stability) of NPs following their administration in the complex environment of biological fluids. Since bioavailability and potential toxicological effects of NPs are dependent on their dispersion state, an ideal coating material provides high colloidal stability for the resultant NPs in salt- and protein-containing media, such as buffer solutions or cell culture media, for their in vitro testing in biological cells and in vivo testing in animal models [120].

Regarding iron oxide NPs, numerous formulations have been studied in both preclinical and clinical settings, and some of them have already been introduced into the market. Besides, there are several FDA (Food and Drug Administration)-approved iron oxide NPs for clinical use, such as Feraheme^®^ (for iron deficiency, Combidex^®^ (U.S.), and Sinerem^®^ (Europe) as magnetic resonance imaging (MRI) agents, Nanotherm^®^ (MagForce) for cancer treatment and Lumirem^®^ as an oral gastrointestinal tract imaging agent.

Feraheme^®^ (ferumoxytol injectable solution) was also approved in Canada (2011) and Europe (2012) and has been clinically used for treating iron-deficiency anemia (IDA). In addition, ferumoxytol has shown great promise for many other biomedical applications, including MRI, drug delivery, oral biofilm treatment, and anti-cancer and anti-inflammatory therapies. For instance, ferumoxytol is being used as an MRI contrast agent in ongoing clinical trials [121].

In 2017, Emtiazi et al. synthesized micro and nanotubes from the self-assembly of FNTs conjugated with FA/MNPs and studied their potential applications for the delivery of the well-known anticancer therapeutic 5-fluorouracil (5-FU). The conjugation was performed by covalent linkage of the carboxylic groups on FA/MNPs with the amine groups on FNTs (using the *N*-hydroxysuccinimide/carbodiimide (NHS/EDC) chemistry). 5-FU was loaded in FNT hydrogels, exhibiting a controlled and slow release, specifically within the first 2 h. This nanocomposite combined biocompatibility and biofunctionality in a single platform with a high selectivity towards MCF-7 cancer cells [122].

Multi-drug resistance (MDR) is a critical contributor to over 90% of deaths of patients in traditional cancer chemotherapy [123]. Cancer cells show MDR due to either acquired defense behaviors or inherent mechanisms, including enhanced drug efflux and DNA repair capacity, elevated xenobiotics metabolism, and even genetic factors (e.g., epigenetic alterations and gene mutations) [124]. Based on these diverse modes of MDR development, it is imperative to introduce novel methods to overcome MDR mechanisms and enhance the survival rate.

In the past decade, RNA interference (RNAi) technology has been introduced as a potential alternative to conventional chemotherapy because this kind of gene therapy can inhibit almost every single protein expression of the target cancer cells [125], which can eliminate the MDR phenotype in cancer cells. This unique technology has demonstrated a high specificity towards the target cells, and therefore, it is able to reduce the most common side effects of chemotherapy. For the clinical applications of small interfering RNA (siRNA), however, there are some limitations, including the low transport efficiency of siRNA and their instability/high degradation rate [126]. Injectable hydrogels have been developed to address these challenges, having minimal adverse effects and highly controlled delivery of different cargos [127], which could also increase patient comfort with a single injection of the drugs.

To achieve this aim, DNA has been commonly applied as a crosslinker in hydrogels, having unique features such as structural rigidity/designability and excellent targeting capability [128]. Moreover, DNA crosslinked hydrogels provide very high injectability and thixotropic abilities due to their hydrogen bond formation with complementary DNA sequences [129].

In 2023, Chen et al. synthesized an injectable hydrogel nanocomposite for the co-delivery of the anticancer drug doxorubicin and MDR-targeted siRNAs for its potential application for combined gene- and chemotherapy. Specifically, tetra-armed PEG served as the backbone of the hydrogel nanostructure. The authors used tetra-armed PEGs to prepare the hydrogel, and complementary DNA sequences were cross-linked into the hydrogel matrix by hydrogen bonds between the DNA–base pairing. To enhance the mechanical properties of this hybrid hydrogel, laponite nanoclay was incorporated into the gel matrix by physical interaction. Moreover, both nanoclay and DNA sequences improved the loading capacity of the nanocomposite for positively charged doxorubicin by intercalating within the interlayer spaces of nanoclay and the DNA structure. Then, MDR-targeted siRNAs, which were previously complexed with the membrane-permeable peptides with stearyl-octaarginine (STR-R8), were incorporated within the hydrogels. To control the degradation of this composite, MMP-2 cleavable peptide sequences were also conjugated to DNA sequences and tetra-armed PEG. By the upregulated expression of the MMP-2 tumor-associated enzyme, this hydrogel nanocomposite was degraded and released dsDNA/doxorubicin, laponite/doxorubicin, TR-R8/siRNA, and complexes. Doxorubicin released from the nanoclay complexes first damaged the target tumor (breast cancer) without the MDR effect. Then, the cancer cells endocytosed STR-R8/siRNA, followed by doxorubicin release from dsDNA/doxorubicin, to eliminate MDR cancer cells. This composite was directly injected into the tumor site thanks to its in situ gelation and degradability properties, which demonstrate its potential capacity in targeted cancer therapy with low side effects. The authors suggested that this DDS nanocomposite can deal with the MDR effect and potentially prevent tumor metastasis [130].

In chemotherapy, potent pharmaceutics are administrated in specific intervals at high concentrations, which inevitably causes their unselective distribution in the whole body and sometimes brings irreversible damage to healthy tissues [131,132,133]. Localized drug delivery is an alternative methodology to ensure highly localized drug dosages at the target sites, decreasing the unselective toxicity on non-cancerous organs [134,135,136]. This aim is perfectly achievable using hydrogel and NP-DDSs [137,138], and poly(lactic-co-glycolic acid) (PLGA) NPs have been extensively studied for this aim due to their high biocompatibility and tunable degradability [139,140]. Their practical applications, however, have been restricted due to their quick diffusion into the surrounding tissues, which can be modified by the fabrication of hydrogel-PLGA nanocomposites.

As mentioned in the introduction section, the combination of NPs with peptide-based hydrogels can reinforce the fragile structure of hydrogels and enhance their in vivo stability, as well as tunable drug release [141,142,143,144]. Simultaneously, the peptide hydrogel serves as a fixing scaffold for NPs at a local site to prevent their uncontrolled diffusion. So, these nanocomposites benefit both nano- and gel-type materials by incorporating multiple drugs having different pharmaceutical characters in the various domains of the hydrogel nanocomposites for independent tuning of their release patterns [145,146].

In 2020, Wu et al. reported the synthesis of a novel and injectable peptide-based hydrogel nanocomposite for the controlled co-delivery of cisplatin and irinotecan (cisplatin/Peptide@NP/irinotecan), composed of an inner part of irinotecan-loaded PLGA NPs and an outer section of cisplatin-loaded hydrogel to provide the differential release of irinotecan and cisplatin. Due to the presence of PLGA, this nanocomposite exhibited superior mechanical properties than pristine hydrogel and cisplatin/peptide. As mentioned above, cisplatin and irinotecan have shown synergistic anticancer effects on various cancers, e.g., esophageal adenocarcinoma, glioma, and non-small cell lung cancer (NSCLC) [87,147,148,149], especially when irinotecan was used after cisplatin [86] which results in higher therapeutic effects and lower side effects. This injectable nanocomposite demonstrated a longer retention time in mice compared to the individual formulations (when subcutaneously administered). The in vitro drug release profiles showed a sustained release of irinotecan and a fast release of cisplatin before irinotecan, with a synergic anticancer effect against NSCLC A549. Thus, this study provides a template technology for hydrogel nanocomposites with tunable drug release, enhanced mechanical stability, and improved anticancer efficacy through the synergism of multiple drugs [150].

As mentioned above, combination chemotherapy is generally much more effective than single-drug chemotherapy, which has been well-documented in clinical settings [151,152,153,154]. Current combined chemotherapy is based on the sequential injections of different drugs, requiring long-term patient hospitalization and precise monitoring by trained specialists, which is highly uncomfortable for patients and increases medical costs. It is predicted that next-generation chemotherapy will rely on smart DDSs, and in this regard, hydrogels and nanomaterials (e.g., metal and metal oxide NPs, micelles, liposomes, nanofibers, and polymeric NPs) are promising candidates possessing unique advantages, such as drug capacity and control of release [155,156,157,158,159,160,161].

Graphene oxide (GO) nanocarriers have been introduced as suitable DDSs because of their high specific surface area (up to 500 m^2^ g^−1^) [162], suitable for loading large amounts of poorly bioavailable drugs, e.g., anthracyclines, taxanes, and camptothecan analogs [163,164,165,166,167,168,169]. GO surfaces have numerous hydroxyl groups, epoxides, and carboxylic acids that are suitable functional groups for conjugating other targeting and stabilizing molecules to enhance therapeutic efficiency and prolong drug circulation time [170,171].

Regarding peptide-based hydrogels, “designer” peptides have been used to synthesize hydrogel-based DDSs because of their inherent biocompatibility, tenability, biodegradability, and fast gelation via hierarchal self-assembly [172]. As a commonly used designer peptide, Max8 sequence (VKVKVKVKVDPPTKVEVKVKV-NH_2_, DP: d-proline) is a stimuli-responsive peptide which shows stability at low ionic strength and self-assembles into 3.2 nm diameter *β*-hairpin nanofibers in physiological conditions [173]. Branco et al. showed that Max8-hydrogel has little resistance to the transport of both large and small molecules (e.g., fluorescein–dextran conjugates) at low gel fractions (0.5–2 *w*/*w*) [174]. In combination chemotherapy, the limited control over the relative flux of multiple diffusing drugs restricts their practical in vivo applications. Hydrogel nanocomposite systems containing nanocarriers embedded in the peptide hydrogel have shown great promise for precise delivery of multiple cargos [175,176,177,178].

In 2020, Schneible et al. developed a new nanocomposite comprising doxorubicin-loaded modified-GO NPs embedded in a gemcitabine-loaded Max8 hydrogel. The synergistic effect of doxorubicin and gemcitabine was extensively studied in terms of pharmacokinetic and molar ratio (gemcitabine/doxorubicin > 1) [179,180,181,182] with the optimal synergic effect at 10:1 ratio when doxorubicin was being administered after gemcitabine [183]. The authors studied the surface modification of GO NPs at different conditions to tune their hydrophobicity and surface charge; then, doxorubicin loading and release at different ionic strengths and pHs were investigated. Interestingly, surface modification with tris(2-aminoethyl) amine (TREN) resulted in high drug loading (0.2–0.6 mg doxorubicin/mg GO) and showed an initially fast release of 18.9% of the drug (within 72 h), followed by a sustained release (31.4% over 4 weeks). Using molecular dynamics simulations, the doxorubicin/TREN-GO interaction at different conditions to gain molecular-level insight into the release/adsorption behavior of the drug in the TREN-GO system was also studied. Afterward, DOX–TREN-GO NPs were embedded in a gemcitabine/Max8 hydrogel and successfully tested on a triple-negative breast cancer cell line (MDA-MB-231). The composite DDS demonstrated a high synergic index of 0.093 ± 0.001, significantly lower than the free doxorubicin-gemcitabine combination (CI = 0.396 ± 0.034) at the same 1:10 molar ratio and concentration [184].

In vivo studies of NPs have shown their rapid clearance via the reticuloendothelial system (RES) and accumulation at the tumor site [185] due to the enhanced permeability and retention (EPR) effect because of the leakiness of tumor blood vessels [186,187]. Nano-DDSs show different mechanisms to enter cells, such as endocytosis, which can be divided into five distinct subgroups (micropinocytosis and phagocytosis, clathrin- and caveolin-independent endocytosis, clathrin-dependent endocytosis, and caveolin-dependent endocytosis). Alternatively, nano-carriers can cross the plasma membrane of cells via physical or biochemical ways to directly enter the cytoplasm by lipid fusion electroporation, microinjection, or translocation [188]. NP features (e.g., surface chemistry, morphology, size, chemical nature, charge) affect their interaction and mechanism of cell uptake [189]. Another advantage of nano-DDSs is that they can simultaneously carry multiple biologically relevant molecules (nucleic acids, organic/metallic drugs, and contrast agents) [190,191,192]. Nano-DDSs can safeguard the loaded bioactive molecules from potential degradation/inactivation in the bloodstream and guarantee the safe reaching of these nano-carriers to their targets, together with the controlled release of drugs to enhance in vivo therapeutic efficacy [193]. Moreover, some poorly hydrophilic drugs (e.g., danazol, paclitaxel, and naproxen) can be formulated using nanocarriers without requiring non-biocompatible organic solvents for their solubilization [194], which provides improved in vivo pharmacodynamic and pharmacokinetic properties of the drug and reduced toxicity. Regarding doxorubicin, for instance, in 1995, the Food and Drug Administration (FDA) introduced a liposomal formulation (commercially known as Doxil^®^/Caelyx^®^) for several tumors such as metastatic breast cancer with cardiac risk [195]. This liposomal formulation can significantly reduce cardiotoxicity and myelosuppression of doxorubicin and enhance its pharmaceutical efficiency due to the different biodistribution of the formulated drug [196,197]. There are currently a few other commercialized drug nano-carriers [198], such as Eligard^®^ (based on polymeric PLGA (poly(lactic-co-glycolic acid), for prostate cancer therapy), Abraxane^®^ and Genexol PM^®^ (for paclitaxel delivery, metastatic breast cancer), and one for Irinotecan vehiculation (Onivyde^®^, for pancreatic cancer) [199].

Nanogels (NGs) have been studied as novel biocompatible DDSs, specifically for the in vivo delivery of contrast agents and therapeutics [200,201]. Nanogels are nano-sized supramolecular assemblies, having an internal hydrogel-like core which is stabilized by an exterior surfactant shell, obtained from the submicronization of macroscopic hydrogels or peptide sequences. Because of their biodegradability, biocompatibility, and easy synthesis method (mild pH and temperature values), peptide-based nanogels are attractive platforms for developing DDSs. Similarly to micelles and liposomes, NGs are injectable and compatible with accumulation and prolonged bloodstream circulation, and different targeting molecules (e.g., peptides, antibodies, or small organic molecules) can be attached to their surface to recognize the site of action. Unlike micelles and liposomes, NGs resemble hydrogels (due to their inner matrix) with their entangled fibrillary and porous network, which can accommodate a large quantity of water and establish non-covalent interactions between their peptide moieties (aromatic and aliphatic groups of amino acid segments), which is probably the most important feature of peptide-NGs because they can be easily tuned for modifying their loading and release properties by simply changing their primary peptide sequence.

In 2020, Smaldone et al. introduced a highly stable peptide-NG formulation [202], which was synthesized using Nα-9-fluorenylmethoxycarbonyl-diphenylalanine and Fmoc-FF (Fmoc-Phe-Phe-OH, a well-studied low molecular weight hydrogelator), forming self-assembling hydrogels under physiological conditions [203] with the capability of doxorubicin encapsulation [204,205]. The authors evaluated the cytotoxicity of the unloaded NGs on a panel of breast cancer cell lines. By the treatment with unloaded NGs, one of the tested cell lines was more affected than the others, so it was found that this cargo-independent cell-specific cytotoxicity is due to the specificity of the machinery used by Fmoc-FF NGs, which shows a selectivity towards the cancer cell lines overexpressing the protein caveolin1 and efficiently performing caveolae-mediated endocytosis.

Anticancer immunotherapy is based on helping the immune system (using synthetic or natural regents) to specifically damage cancer cells, a promising anticancer treatment with huge potential. As a type of immunotherapy, checkpoint inhibitor therapy (ICT) blocks proteins that prevent the immune system from attacking cancer cells. The programmed cell death-1 (PD-1) and programmed death ligand-1 (PD-L1) have exhibited a potent response in various tumors such as triple-negative breast cancer (TNBC); however, only a small portion of TNBC patients respond to ICT because of the immunosuppressive tumor microenvironment (TME) and immunologically “cold” tumors, which later show low mutational load, lack of T-cell infiltration, deficient PD-L1, and low MHC I expressions. To improve the antitumor efficacy, complementary therapies are necessary to remold TME. For enhancing PD-1/L1 effectiveness, the modulation of T cells is desirable (to make an inflamed “hot tumor”) [206,207,208].

Immunogenic cell death (ICD) mechanism triggers the antitumor immune response, which is well known to improve/modify the low immunogenicity of “cold” tumors by releasing damage-associated molecular patterns (DAMPs) [209], such as adenosine triphosphate (ATP) secretion, calreticulin (CRT) surface exposure, and high mobility group box 1 protein (HMGB1), followed by the maturation of antigen-presenting dendritic cells (DCs) and inducing a cascade process leading to antigen-specific T-cell infiltration [210]. Also, the binding of TLR4 to HMGB1 triggers inflammatory responses as well [211]. These in situ vaccine-like phenomena can induce immune responses to ease the transformation of “cold tumors” into “hot tumors” and eventually reshape the immunosuppressive microenvironment and remove cancer cells [212].

Previous research has shown that some therapeutics (e.g., radio-/photodynamic therapy, hyperthermia, and some types of chemotherapy [213]) can initiate ICD, which makes the tumor accessible to the immune system. Chemotherapy-induced ICD plays a key role in improving immunotherapy, and doxorubicin chemo-drug has been frequently used for various malignancies, stimulating ICD-induced immunity [214]. However, this doxorubicin immunogenicity is weak by itself, and tumor relapse can often be detected in clinical cases [215].

Traditional Chinese medicine has exhibited great potential for introducing biocompatible cancer adjuvants. Ginsenoside Rg3 is a steroidal saponin obtained from Panax ginseng, which has shown various antitumor effects and immune-modulatory activities. More importantly, the combination of well-known chemotherapeutics (e.g., doxorubicin, docetaxel, and paclitaxel) with Rg3 has received great attention for significant antitumor activities in different kinds of malignant tumors [216]. Rg3 can also improve the ICD effect stimulated by doxorubicin and activated by the immunity system in acute myeloid leukemia (AML) mice [217]. However, its poor solubility and lack of targeting ability restrict Rg3 penetration into tumors, thus decreasing the outcome of combination doxorubicin-Rg3 therapy.

Chitosan (CS) is a natural polysaccharide with high biodegradability and biocompatibility [218], possessing free –NH_2_ and –OH functional groups in its structure, which are amenable to chemical modifications, for its biological applications [219], including lysosomal escape, targeted-drug delivery and TME response [220,221,222]. It is known that low drug penetration leads to the failure of immunotherapeutic treatments (e.g., TNBC) [223,224], so deep tumor penetration is required in chemotherapy. Cell-penetrating peptides (CPPs) may solve this issue, as they are able to transfer NPs into the cell and improve their curative effectiveness [225].

Cell-penetrating peptides (CPPs) are short peptides (fewer than 30 amino acids) that have been used in preclinical research over the last three decades. Since they facilitate drug or CPP/cargo transport across the plasma membrane (through endocytosis or by perturbation of the lipid bilayer of the cell membrane), they have potential applications in disease diagnosis, including cancer, inflammation, central nervous system disorders, diabetes, otologic and ocular disorders. However, there are no FDA-approved CPPs or CPP/cargo complexes because many issues still need to be addressed before their clinical translation [226,227,228].

**Figure 4:**
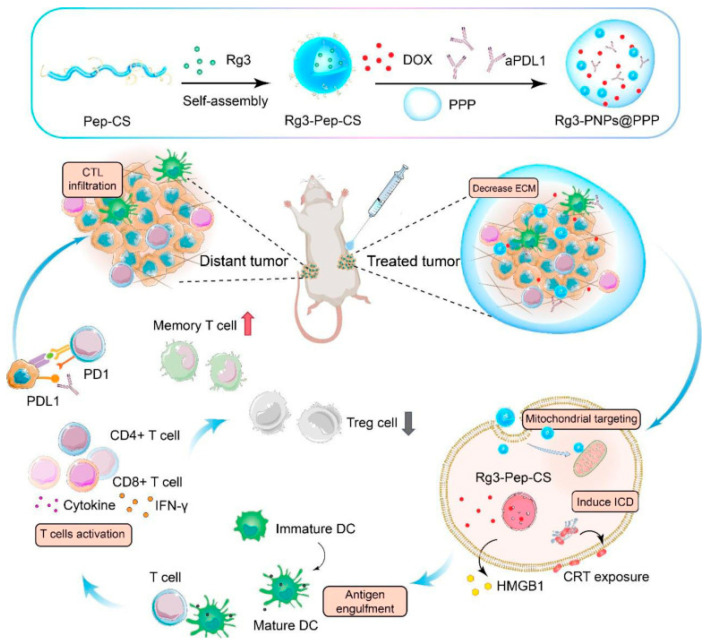
Schematic illustration of a thermo-sensitive hydrogel formation and inhibition of local chemotherapy combined with immunotherapy on abscopal tumors. Reprinted from [230], an open access article distributed under the terms of the Creative Commons CC BY license. Copyright © 2022.

To minimize the short circulation time and systemic toxicity of doxorubicin, cell-penetrating peptide (R6F3)-modified NPs (PNPs) and chitosan loaded with ginsenoside Rg3 (Rg3) were synthesized using the self-assembly technique, followed by co-encapsulation with doxorubicin-based on thermo-sensitive hydrogel [229]. In a recent work, Wu et al. studied localized chemo-immunotherapy using this thermo-sensitive hydrogel nanocomposite to help the anti-tumor immunotherapeutic efficacy for 4T1 tumor (Figure 4) as a typical TNBC model [230]. The targeted delivery of Rg3 by chitosan and transmembrane peptides can degrade the tumor’s extracellular matrix and decrease its solid stress. Moreover, due to the presence of loaded R6F3, Rg3 could dramatically penetrate into tumor cells, followed by targeting mitochondria due to positively charged NPs, thus reinforcing the ICD effect triggered by doxorubicin. Thus, abundant tumor cell debris was detected, with subsequent T cell activation and DC maturation. This methodology decreased the tumor volume in the orthotopic 4T1 model and significantly prolonged its survival time, demonstrating a reliable approach to improve the checkpoint-blocking therapy for 4T1 tumors and converting immune “cold” 4T1 into “hot” tumors.

Other publications on peptide-based hydrogel nanocomposites for biological applications are summarized in Table 3.

## 3. Conclusions

Traditional chemotherapy is still restricted by the low effectiveness and systemic toxicities of drugs. Due to the high recurrence rates of some types of cancers, tumor resection is not the most reliable choice, and therefore, advanced DDSs are required. Hydrogel nanocomposites have shown to be great candidates to enhance the therapeutic efficacy of anticancer drugs. Due to their biodegradability, biocompatibility, and stimuli responsiveness, they are excellent platforms for either passive or targeted drug delivery applications. Combining both peptide-based hydrogels and NPs in a single composite, they have successfully demonstrated the ability to target cancer cells and tumors with high selectivity without compromising healthy tissues. Proof-of-concept studies have shown successful in vivo models. However, for the use of hydrogel nanocomposites in clinical trials, they should have some basic properties: (1) excellent mechanical strength and injectability to enhance in vivo drug stability as well as in situ protection/retention of drugs; (2) strong drug–carrier interactions to guarantee controlled and sustained drug release; (3) tunability of the structure tunable for having different release profiles based on the type of drug; and (4) capacity/ability to load several therapeutic components inside the hydrogel matrix to achieve a synergic efficacy and multifunctionality.

The design of smart hydrogel nanocomposites based on peptide networks and NPs is still a challenging field, with a broad range of physicochemical and therapeutic properties to be controlled and understood. So, more computational and experimental studies are necessary to rationalize their dynamic behavior and relevant interactions with drugs, cells, and tissues to develop more effective formulations that may maximize the selectivity and efficiency of chemotherapy. From a futuristic point of view, we believe that peptide-based hydrogel nanocomposites will be further developed as novel DDSs to maximize the selectivity and efficiency of the chemotherapy.

While significant advances have been recently made in optimizing hydrogel nanocomposites, there are still some challenges for their clinical application in drug delivery. For instance, foreign body reactions frequently cause collagenous capsule formation, which restricts the performance of implantable nanocomposites. To address this challenge, ultra-low-fouling zwitterionic hydrogels have been recently developed to resist capsule formation.

The in vivo safety of hydrogel nanocomposites is another main crucial issue which makes product development challenging; therefore, it is vital to gradually decrease the threshold through multidisciplinary collaborations between chemists, materials scientists, biologists, and clinicians in order to define the future role of peptide-based hydrogel nanocomposites in the field of anti-cancer drug delivery.

## Figures and Tables

**Table 1:** Examples of nanocomposite hydrogels with properties that are triggered or enhanced by nanoparticles for DDS applications. Reprinted with permission from [38]. Copyright 2015 American Chemical Society.

Benefits	Nanoparticles	Ref.
Controlled drug release under electrical stimuli	Carbon NPs	[38,45]
Controlled drug release under magnetic stimuli	Iron oxide magnetic NPs	[38,46]
Controlled drug release under light stimuli	Carbon NPs	[38,47]

**Table 2:** Summary of the DDS applications, advantages, and disadvantages associated with the peptide-based hydrogels loaded with inorganic nanoparticles. The disadvantages can be transversal to other composites [50].

Hydrogel-Inorganic Nanoparticle Composite	Advantages	Disadvantages
Iron oxide magnetic NPs [69,70,71]	Synergy with magnetic hyperthermia Magnetoresponse MRI contrast	Requires screening functionalization to achieve co-assembly
Gold/silver NPs [72]	Low-cost sensors Synthesis in situ Facile synthesis and tunability Synergy with photothermia /photodynamic therapy	Heterogeneous dispersion Uncontrolled release Challenging reproducibility of in situ synthesis

**Table 3:** Other hydrogel nanocomposites used for biological applications.

Hydrogel Nanocomposite	Application	Ref.
Oxidized carbon nanotubes GO + (Fmoc-Phe-OH/Fmoc-Tyr(Bzl)-OH Fmoc/Tyr-OH/Fmoc-Tyr(Bzl)-OH) hydrogel	drug delivery	[231]
Graphene quantum dots + (Amoc-Phe-OH Amoc-Tyr-OH) hydrogel	drug delivery	[232]
GO + (Py-Gly-Ala-Gly-Ala-Gly-Tyr-OH) hydrogel	drug delivery	[233]
Fmoc-FFpY-hydrogel assembled from enzyme-adsorbed mesoporous silica nanostructures	thermo-responsive doxorubicin release	[234]
Fmoc–FFpY hydrogel nanocomposite containing silica NPs functionalized covalently by alkaline phosphatase	N/A	[235]
graphene oxide as nano-filler for the reinforcement of FEFKFEFK (β-sheet forming self-assembling peptide)	intervertebral disc repair applications	[236]
self-assembling a motif-specific peptide molecule (LLVFGAKMLPHHGA) containing graphene foam	matrices for drug delivery or bone tissue engineering	[237]
RGDAEAKAEAKYWYAFAEAKAEAKRGD-hydrogel-graphene quantum dots	targeting and imaging of tumor cells	[238]
self-assembly of an amphiphilic peptide (APP) into a nanochain with subsequent chemical crosslinking of NIR-II Ag_2_S QDs	ultrasensitive Detection of Peritoneal Metastasis	[239]

## Data Availability

Not applicable.

## References

[1] Voycheva C., Slavkova M., Popova T., Tzankova D., Stefanova D., Tzankova V., Ivanova I., Tzankov S., Spassova I., Kovacheva D. (2023). Thermosensitive Hydrogel-Functionalized Mesoporous Silica Nanoparticles for Parenteral Application of Chemotherapeutics. Gels.

[2] Pulumati A., Pulumati A., Dwarakanath B.S., Verma A., Papineni R.V.L. (2023). Technological Advancements in Cancer Diagnostics: Improvements and Limitations. Cancer Rep..

[3] Cerra S., Dini V., Salamone T.A., Hajareh Haghighi F., Mercurio M., Cartoni A., Del Giudice A., Marsotto M., Venditti I., Battocchio C. (2023). Acrylates-Based Hydrophilic Co-Polymeric Nanobeads as Nanocarriers for Imaging Agents. Colloids Surf. A Physicochem. Eng. Asp..

[4] Shen J., Zhang J., Wu W., Banerjee P., Zhou S. (2023). Biocompatible Anisole-Nonlinear PEG Core–Shell Nanogels for High Loading Capacity, Excellent Stability, and Controlled Release of Curcumin. Gels.

[5] Goel M., Mackeyev Y., Krishnan S. (2023). Radiolabeled Nanomaterial for Cancer Diagnostics and Therapeutics: Principles and Concepts. Cancer Nanotechnol..

[6] Alhussan A., Jackson N., Eaton S., Santos N.D., Barta I., Zaifman J., Chen S., Tam Y.Y.C., Krishnan S., Chithrani D.B. (2022). Lipid-Nanoparticle-Mediated Delivery of Docetaxel Prodrug for Exploiting Full Potential of Gold Nanoparticles in the Treatment of Pancreatic Cancer. Cancers.

[7] Luo D., Wang X., Walker E., Springer S., Ramamurthy G., Burda C., Basilion J.P. (2022). Targeted Chemoradiotherapy of Prostate Cancer Using Gold Nanoclusters with Protease Activatable Monomethyl Auristatin E. ACS Appl. Mater. Interfaces.

[8] Elballa W., Schwinghamer K., Ebert E., Siahaan T.J., Jois S.D. (2022). Peptides and Their Delivery to the Brain BT—Peptide Therapeutics: Fundamentals of Design, Development, and Delivery.

[9] Kargari Aghmiouni D., Khoee S. (2023). Dual-Drug Delivery by Anisotropic and Uniform Hybrid Nanostructures: A Comparative Study of the Function and Substrate&ndash;Drug Interaction Properties. Pharmaceutics.

[10] Hajareh Haghighi F., Mercurio M., Cerra S., Salamone T.A., Bianymotlagh R., Palocci C., Romano Spica V., Fratoddi I. (2023). Surface Modification of TiO2 Nanoparticles with Organic Molecules and Their Biological Applications. J. Mater. Chem. B.

[11] Asad S., Jacobsen A.-C., Teleki A. (2022). Inorganic Nanoparticles for Oral Drug Delivery: Opportunities, Barriers, and Future Perspectives. Curr. Opin. Chem. Eng..

[12] Anselmo A.C., Mitragotri S. (2021). Nanoparticles in the Clinic: An Update Post COVID-19 Vaccines. Bioeng. Transl. Med..

[13] Khosravian P., Shafiee Ardestani M., Khoobi M., Ostad S.N., Dorkoosh F.A., Akbari Javar H., Amanlou M. (2016). Mesoporous Silica Nanoparticles Functionalized with Folic Acid/Methionine for Active Targeted Delivery of Docetaxel. Onco. Targets Ther..

[14] Quan Q., Xie J., Gao H., Yang M., Zhang F., Liu G., Lin X., Wang A., Eden H.S., Lee S. (2011). HSA Coated Iron Oxide Nanoparticles as Drug Delivery Vehicles for Cancer Therapy. Mol. Pharm..

[15] Mitchell M.J., Billingsley M.M., Haley R.M., Wechsler M.E., Peppas N.A., Langer R. (2021). Engineering Precision Nanoparticles for Drug Delivery. Nat. Rev. Drug Discov..

[16] Norouzi M., Yathindranath V., Thliveris J.A., Kopec B.M., Siahaan T.J., Miller D.W. (2020). Doxorubicin-Loaded Iron Oxide Nanoparticles for Glioblastoma Therapy: A Combinational Approach for Enhanced Delivery of Nanoparticles. Sci. Rep..

[17] Musial J., Krakowiak R., Mlynarczyk D.T., Goslinski T., Stanisz B.J. (2020). Titanium Dioxide Nanoparticles in Food and Personal Care Products—What Do We Know about Their Safety?. Nanomaterials.

[18] Bhullar S., Goyal N., Gupta S. (2022). A recipe for optimizing TiO_2_ nanoparticles for drug delivery applications. OpenNano.

[19] Hussein H.A., Abdullah M.A. (2022). Novel Drug Delivery Systems Based on Silver Nanoparticles, Hyaluronic Acid, Lipid Nanoparticles and Liposomes for Cancer Treatment. Appl. Nanosci..

[20] Sibuyi N.R.S., Moabelo K.L., Fadaka A.O., Meyer S., Onani M.O., Madiehe A.M., Meyer M. (2021). Multifunctional Gold Nanoparticles for Improved Diagnostic and Therapeutic Applications: A Review. Nanoscale Res. Lett..

[21] Koushki K., Keshavarz Shahbaz S., Keshavarz M., Bezsonov E.E., Sathyapalan T., Sahebkar A. (2021). Gold Nanoparticles: Multifaceted Roles in the Management of Autoimmune Disorders. Biomolecules.

[22] Gerosa C., Crisponi G., Nurchi V.M., Saba L., Cappai R., Cau F., Faa G., Van Eyken P., Scartozzi M., Floris G. (2020). Gold Nanoparticles: A New Golden Era in Oncology?. Pharmaceuticals.

[23] Li X., Zhang Y., Liu G., Luo Z., Zhou L., Xue Y., Liu M. (2022). Recent Progress in the Applications of Gold-Based Nanoparticles towards Tumor-Targeted Imaging and Therapy. RSC Adv..

[24] Yang Z., Wang D., Zhang C., Liu H., Hao M., Kan S., Liu D., Liu W. (2022). The Applications of Gold Nanoparticles in the Diagnosis and Treatment of Gastrointestinal Cancer. Front. Oncol..

[25] Kaphle A., Jayarathna S., Moktan H., Aliru M., Raghuram S., Krishnan S., Cho S.H. (2023). Deep Learning-Based TEM Image Analysis for Fully Automated Detection of Gold Nanoparticles Internalized Within Tumor Cell. Microsc. Microanal..

[26] Jackson N., Hill I., Alhussan A., Bromma K., Morgan J., Abousaida B., Zahra Y., Mackeyev Y., Beckham W., Herchko S. (2023). Dual Enhancement in the Radiosensitivity of Prostate Cancer through Nanoparticles and Chemotherapeutics. Cancer Nanotechnol..

[27] Raghuram S., Mackeyev Y., Symons J., Zahra Y., Gonzalez V., Mahadevan K.K., Requejo K.I., Liopo A., Derry P., Zubarev E. (2022). Uncloaking Cell-Impermeant Gold Nanorods via Tumor Microenvironmental Cathepsin B Facilitates Cancer Cell Penetration and Potent Radiosensitization. Biomaterials.

[28] Rauta P.R., Mackeyev Y., Sanders K., Kim J.B.K., Gonzalez V.V., Zahra Y., Shohayeb M.A., Abousaida B., Vijay G.V., Tezcan O. (2023). Pancreatic Tumor Microenvironmental Acidosis and Hypoxia Transform Gold Nanorods into Cell-Penetrant Particles for Potent Radiosensitization. Sci. Adv..

[29] Zhao C., Man T., Xu X., Yang Q., Liu W., Jonas S.J., Teitell M.A., Chiou P.-Y., Weiss P.S. (2020). Photothermal Intracellular Delivery Using Gold Nanodisk Arrays. ACS Mater. Lett..

[30] Anselmo A.C., Mitragotri S. (2019). Nanoparticles in the Clinic: An Update. Bioeng. Transl. Med..

[31] Da Silva C.G., Peters G.J., Ossendorp F., Cruz L.J. (2017). The Potential of Multi-Compound Nanoparticles to Bypass Drug Resistance in Cancer. Cancer Chemother. Pharmacol..

[32] Binaymotlagh R., Chronopoulou L., Palocci C. (2023). Peptide-Based Hydrogels: Template Materials for Tissue Engineering. J. Funct. Biomater..

[33] Binaymotlagh R., Chronopoulou L., Haghighi F.H., Fratoddi I., Palocci C. (2022). Peptide-Based Hydrogels: New Materials for Biosensing and Biomedical Applications. Materials.

[34] Chronopoulou L., Binaymotlagh R., Cerra S., Haghighi F.H., Di Domenico E.G., Sivori F., Fratoddi I., Mignardi S., Palocci C. (2023). Preparation of Hydrogel Composites Using a Sustainable Approach for in situ Silver Nanoparticles Formation. Materials.

[35] Binaymotlagh R., Del Giudice A., Mignardi S., Amato F., Marrani A.G., Sivori F., Cavallo I., Di Domenico E.G., Palocci C., Chronopoulou L. (2022). Green in situ Synthesis of Silver Nanoparticles-Peptide Hydrogel Composites: Investigation of Their Antibacterial Activities. Gels.

[36] Chen Q., Wang C., Zhang X., Chen G., Hu Q., Li H., Wang J., Wen D., Zhang Y., Lu Y. (2019). In situ Sprayed Bioresponsive Immunotherapeutic Gel for Post-Surgical Cancer Treatment. Nat. Nanotechnol..

[37] Wang Y., Zhang X., Wan K., Zhou N., Wei G., Su Z. (2021). Supramolecular Peptide Nano-Assemblies for Cancer Diagnosis and Therapy: From Molecular Design to Material Synthesis and Function-Specific Applications. J. Nanobiotechnol..

[38] Merino S., Martín C., Kostarelos K., Prato M., Vázquez E. (2015). Nanocomposite Hydrogels: 3D Polymer–Nanoparticle Synergies for On-Demand Drug Delivery. ACS Nano.

[39] Choi S., Choi Y.J., Jang M.-S., Lee J.H., Jeong J.H., Kim J. (2017). Supertough Hybrid Hydrogels Consisting of a Polymer Double-Network and Mesoporous Silica Microrods for Mechanically Stimulated On-Demand Drug Delivery. Adv. Funct. Mater..

[40] Wu Y., Wang H., Gao F., Xu Z., Dai F., Liu W. (2018). An Injectable Supramolecular Polymer Nanocomposite Hydrogel for Prevention of Breast Cancer Recurrence with Theranostic and Mammoplastic Functions. Adv. Funct. Mater..

[41] Liu C., Guo X., Ruan C., Hu H., Jiang B.-P., Liang H., Shen X.-C. (2019). An Injectable Thermosensitive Photothermal-Network Hydrogel for near-Infrared-Triggered Drug Delivery and Synergistic Photothermal-Chemotherapy. Acta Biomater..

[42] Wu H., Song L., Chen L., Zhang W., Chen Y., Zang F., Chen H., Ma M., Gu N., Zhang Y. (2018). Injectable Magnetic Supramolecular Hydrogel with Magnetocaloric Liquid-Conformal Property Prevents Post-Operative Recurrence in a Breast Cancer Model. Acta Biomater..

[43] Cai T., Huo S., Wang T., Sun W., Tong Z. (2018). Self-Healable Tough Supramolecular Hydrogels Crosslinked by Poly-Cyclodextrin through Host-Guest Interaction. Carbohydr. Polym..

[44] McKee J.R., Appel E.A., Seitsonen J., Kontturi E., Scherman O.A., Ikkala O. (2014). Healable, Stable and Stiff Hydrogels: Combining Conflicting Properties Using Dynamic and Selective Three-Component Recognition with Reinforcing Cellulose Nanorods. Adv. Funct. Mater..

[45] Servant A., Methven L., Williams R.P., Kostarelos K. (2013). Electroresponsive Polymer-Carbon Nanotube Hydrogel Hybrids for Pulsatile Drug Delivery in vivo. Adv. Healthc. Mater..

[46] Liu T.Y., Hu S.H., Liu T.Y., Liu D.M., Chen S.Y. (2006). Magnetic-Sensitive Behavior of Intelligent Ferrogels for Controlled Release of Drug. Langmuir.

[47] Cheng Z., Chai R., Ma P., Dai Y., Kang X., Lian H., Hou Z., Li C., Lin J. (2013). Multiwalled Carbon Nanotubes and NaYF_4_:Yb^3+^/Er^3+^ Nanoparticle-Doped Bilayer Hydrogel for Concurrent NIR-Triggered Drug Release and Up-Conversion Luminescence Tagging. Langmuir.

[48] Quazi M.Z., Park N. (2022). Nanohydrogels: Advanced Polymeric Nanomaterials in the Era of Nanotechnology for Robust Functionalization and Cumulative Applications. Int. J. Mol. Sci..

[49] Hajareh Haghighi F., Binaymotlagh R., Chronopoulou L., Cerra S., Marrani A.G., Amato F., Palocci C., Fratoddi I. (2023). Self-Assembling Peptide-Based Magnetogels for the Removal of Heavy Metals from Water. Gels.

[50] Gomes V., Veloso S.R.S., Correa-Duarte M.A., Ferreira P.M.T., Castanheira E.M.S. (2023). Tuning Peptide-Based Hydrogels: Co-Assembly with Composites Driving the Highway to Technological Applications. Int. J. Mol. Sci..

[51] Veloso S.R.S., Andrade R.G.D., Castanheira E.M.S. (2021). Review on the Advancements of Magnetic Gels: Towards Multifunctional Magnetic Liposome-Hydrogel Composites for Biomedical Applications. Adv. Colloid Interface Sci..

[52] Larrañeta E., Stewart S., Ervine M., Al-Kasasbeh R., Donnelly R.F. (2018). Hydrogels for Hydrophobic Drug Delivery. Classification, Synthesis and Applications. J. Funct. Biomater..

[53] Gao W., Zhang Y., Zhang Q., Zhang L. (2016). Nanoparticle-Hydrogel: A Hybrid Biomaterial System for Localized Drug Delivery. Ann. Biomed. Eng..

[54] Bray F., Ferlay J., Soerjomataram I., Siegel R.L., Torre L.A., Jemal A. (2018). Global Cancer Statistics 2018: GLOBOCAN Estimates of Incidence and Mortality Worldwide for 36 Cancers in 185 Countries. CA. Cancer J. Clin..

[55] Xie M., Xu X., Fan Y. (2021). KRAS-Mutant Non-Small Cell Lung Cancer: An Emerging Promisingly Treatable Subgroup. Front. Oncol..

[56] Herbst R.S., Morgensztern D., Boshoff C. (2018). The Biology and Management of Non-Small Cell Lung Cancer. Nature.

[57] Duma N., Santana-Davila R., Molina J.R. (2019). Non–Small Cell Lung Cancer: Epidemiology, Screening, Diagnosis, and Treatment. Mayo Clin. Proc..

[58] Zhu L., Chen L. (2019). Progress in Research on Paclitaxel and Tumor Immunotherapy. Cell. Mol. Biol. Lett..

[59] Jiang X., Zhang B., Zhou Z., Meng L., Sun Z., Xu Y., Xu Q., Yuan A., Yu L., Qian H. (2017). Enhancement of Radiotherapy Efficacy by Pleiotropic Liposomes Encapsulated Paclitaxel and Perfluorotributylamine. Drug Deliv..

[60] Chen Y., Zhu Z., Zhao W., Li L., Ye J., Wu C., Tang H., Lin Q., Li J., Xia Y. (2018). A Randomized Phase 3 Trial Comparing Paclitaxel plus 5-Fluorouracil versus Cisplatin plus 5-Fluorouracil in Chemoradiotherapy for Locally Advanced Esophageal Carcinoma—The ESO-Shanghai 1 Trial Protocol. Radiat. Oncol..

[61] Patel K., Doddapaneni R., Sekar V., Chowdhury N., Singh M. (2016). Combination Approach of YSA Peptide Anchored Docetaxel Stealth Liposomes with Oral Antifibrotic Agent for the Treatment of Lung Cancer. Mol. Pharm..

[62] Peng X.-H., Wang Y., Huang D., Wang Y., Shin H.J., Chen Z., Spewak M.B., Mao H., Wang X., Wang Y. (2011). Targeted Delivery of Cisplatin to Lung Cancer Using ScFvEGFR-Heparin-Cisplatin Nanoparticles. ACS Nano.

[63] Chen Y., Yang J., Fu S., Wu J. (2020). Gold Nanoparticles as Radiosensitizers in Cancer Radiotherapy. Int. J. Nanomed..

[64] Tang J., Fu X., Ou Q., Gao K., Man S.-Q., Guo J., Liu Y. (2018). Hydroxide Assisted Synthesis of Monodisperse and Biocompatible Gold Nanoparticles with Dextran. Mater. Sci. Eng. C.

[65] Sánchez E.G., Quintas A., Pérez-Núñez D., Nogal M., Barroso S., Carrascosa Á.L., Revilla Y. (2012). African Swine Fever Virus Uses Macropinocytosis to Enter Host Cells. PLOS Pathog..

[66] Janczewska M., Szkop M., Pikus G., Kopyra K., Świątkowska A., Brygoła K., Karczmarczyk U., Walczak J., Żuk M.T., Duszak J. (2021). PSMA Targeted Conjugates Based on Dextran. Appl. Radiat. Isot..

[67] Yuan Y., Liao H., Pu Q., Ke X., Hu X., Ma Y., Luo X., Jiang Q., Gong Y., Wu M. (2020). MiR-410 Induces Both Epithelial–Mesenchymal Transition and Radioresistance through Activation of the PI3K/MTOR Pathway in Non-Small Cell Lung Cancer. Signal Transduct. Target. Ther..

[68] Zhang L., Zhou C., Zhou Y., Zhang W., Hu X., Chen M., Hui H., Guo L., Wu C., Zhou J. (2023). P-Y/G@NHs Sensitizes Non-Small Cell Lung Cancer Cells to Radiotherapy via Blockage of the PI3K/AKT Signaling Pathway. Bioorg. Chem..

[69] Nowak B.P., Niehues M., Ravoo B.J. (2021). Magneto-Responsive Hydrogels by Self-Assembly of Low Molecular Weight Peptides and Crosslinking with Iron Oxide Nanoparticles. Soft Matter.

[70] Veloso S.R.S., Martins J.A., Hilliou L., Amorim C.O., Amaral V.S., Almeida B.G., Jervis P.J., Moreira R., Pereira D.M., Coutinho P.J.G. (2020). Dehydropeptide-Based Plasmonic Magnetogels: A Supramolecular Composite Nanosystem for Multimodal Cancer Therapy. J. Mater. Chem. B.

[71] Veloso S.R.S., Silva J.F.G., Hilliou L., Moura C., Coutinho P.J.G., Martins J.A., Testa-Anta M., Salgueiriño V., Correa-Duarte M.A., Ferreira P.M.T. (2021). Impact of Citrate and Lipid-Functionalized Magnetic Nanoparticles in Dehydropeptide Supramolecular Magnetogels: Properties, Design and Drug Release. Nanomaterials.

[72] Wang W., Han R., Tang K., Zhao S., Ding C., Luo X. (2021). Biocompatible Peptide Hydrogels with Excellent Antibacterial and Catalytic Properties for Electrochemical Sensing Application. Anal. Chim. Acta.

[73] Robey R.W., Pluchino K.M., Hall M.D., Fojo A.T., Bates S.E., Gottesman M.M. (2018). Revisiting the Role of ABC Transporters in Multidrug-Resistant Cancer. Nat. Rev. Cancer.

[74] Li W., Zhang H., Assaraf Y.G., Zhao K., Xu X., Xie J., Yang D.-H., Chen Z.-S. (2016). Overcoming ABC Transporter-Mediated Multidrug Resistance: Molecular Mechanisms and Novel Therapeutic Drug Strategies. Drug Resist. Updat..

[75] Park S.B., Goldstein D., Krishnan A.V., Lin C.S.-Y., Friedlander M.L., Cassidy J., Koltzenburg M., Kiernan M.C. (2013). Chemotherapy-Induced Peripheral Neurotoxicity: A Critical Analysis. CA. Cancer J. Clin..

[76] Wang C., Wang J., Zhang X., Yu S., Wen D., Hu Q., Ye Y., Bomba H., Hu X., Liu Z. (2018). In situ Formed Reactive Oxygen Species–Responsive Scaffold with Gemcitabine and Checkpoint Inhibitor for Combination Therapy. Sci. Transl. Med..

[77] Conroy T., Van Laethem J.-L. (2019). Combination or Single-Agent Chemotherapy as Adjuvant Treatment for Pancreatic Cancer?. Lancet Oncol..

[78] Hu Q., Sun W., Wang C., Gu Z. (2016). Recent Advances of Cocktail Chemotherapy by Combination Drug Delivery Systems. Adv. Drug Deliv. Rev..

[79] Conde J., Oliva N., Zhang Y., Artzi N. (2016). Local Triple-Combination Therapy Results in Tumour Regression and Prevents Recurrence in a Colon Cancer Model. Nat. Mater..

[80] Miao L., Guo S., Lin C.M., Liu Q., Huang L. (2017). Nanoformulations for Combination or Cascade Anticancer Therapy. Adv. Drug Deliv. Rev..

[81] Albiges L., Choueiri T., Escudier B., Galsky M., George D., Hofmann F., Lam T., Motzer R., Mulders P., Porta C. (2015). A Systematic Review of Sequencing and Combinations of Systemic Therapy in Metastatic Renal Cancer. Eur. Urol..

[82] Pathak R.K., Dhar S. (2015). A Nanoparticle Cocktail: Temporal Release of Predefined Drug Combinations. J. Am. Chem. Soc..

[83] Weber J.S., Gibney G., Sullivan R.J., Sosman J.A., Slingluff C.L., Lawrence D.P., Logan T.F., Schuchter L.M., Nair S., Fecher L. (2016). Sequential Administration of Nivolumab and Ipilimumab with a Planned Switch in Patients with Advanced Melanoma (CheckMate 064): An Open-Label, Randomised, Phase 2 Trial. Lancet Oncol..

[84] Gainor J.F., Tan D.S.W., De Pas T., Solomon B.J., Ahmad A., Lazzari C., de Marinis F., Spitaleri G., Schultz K., Friboulet L. (2015). Progression-Free and Overall Survival in ALK-Positive NSCLC Patients Treated with Sequential Crizotinib and Ceritinib. Clin. Cancer Res..

[85] Lee M.J., Ye A.S., Gardino A.K., Heijink A.M., Sorger P.K., MacBeath G., Yaffe M.B. (2012). Sequential Application of Anticancer Drugs Enhances Cell Death by Rewiring Apoptotic Signaling Networks. Cell.

[86] Han J.-Y., Lim H.-S., Lee D.H., Ju S.Y., Lee S.Y., Kim H.Y., Park Y.-H., Park C.G., Lee J.S. (2006). Randomized Phase II Study of Two Opposite Administration Sequences of Irinotecan and Cisplatin in Patients with Advanced Nonsmall Cell Lung Carcinoma. Cancer.

[87] Ma J., Maliepaard M., Nooter K., Boersma A.W.M., Verweij J., Stoter G., Schellens J.H.M. (1998). Synergistic Cytotoxicity of Cisplatin and Topotecan or SN-38 in a Panel of Eight Solid-Tumor Cell Lines in vitro. Cancer Chemother. Pharmacol..

[88] Crul M., van Waardenburg R.C.A.,  Beijnen J.H., Schellens J.H.M. (2002). DNA-Based Drug Interactions of Cisplatin. Cancer Treat. Rev..

[89] Adler-Abramovich L., Gazit E. (2014). The Physical Properties of Supramolecular Peptide Assemblies: From Building Block Association to Technological Applications. Chem. Soc. Rev..

[90] Fleming S., Ulijn R. (2014). V Design of Nanostructures Based on Aromatic Peptide Amphiphiles. Chem. Soc. Rev..

[91] Du X., Zhou J., Shi J., Xu B. (2015). Supramolecular Hydrogelators and Hydrogels: From Soft Matter to Molecular Biomaterials. Chem. Rev..

[92] Sato K., Hendricks M.P., Palmer L.C., Stupp S.I. (2018). Peptide Supramolecular Materials for Therapeutics. Chem. Soc. Rev..

[93] Gao J., Zhan J., Yang Z. (2020). Enzyme-Instructed Self-Assembly (EISA) and Hydrogelation of Peptides. Adv. Mater..

[94] Cheetham A.G., Zhang P., Lin Y., Lock L.L., Cui H. (2013). Supramolecular Nanostructures Formed by Anticancer Drug Assembly. J. Am. Chem. Soc..

[95] Cai Y., Shen H., Zhan J., Lin M., Dai L., Ren C., Shi Y., Liu J., Gao J., Yang Z. (2017). Supramolecular “Trojan Horse” for Nuclear Delivery of Dual Anticancer Drugs. J. Am. Chem. Soc..

[96] Zhao H., Xu J., Wan J., Geng S., Li H., Peng X., Fu Q., He M., Zhao Y., Yang X. (2017). Cisplatin-Directed Coordination-Crosslinking Nanogels with Thermo/PH-Sensitive Triblock Polymers: Improvement on Chemotherapic Efficacy via Sustained Release and Drug Retention. Nanoscale.

[97] Li Y., Lu H., Liang S., Xu S. (2019). Dual Stable Nanomedicines Prepared by Cisplatin-Crosslinked Camptothecin Prodrug Micelles for Effective Drug Delivery. ACS Appl. Mater. Interfaces.

[98] Wu C., Liu J., Zhai Z., Yang L., Tang X., Zhao L., Xu K., Zhong W. (2020). Double-Crosslinked Nanocomposite Hydrogels for Temporal Control of Drug Dosing in Combination Therapy. Acta Biomater..

[99] Wang Y., Wang K., Zhao X., Xu X., Sun T. (2023). Influence of PH on the Self-Assembly of Diphenylalanine Peptides: Molecular Insights from Coarse-Grained Simulations. Soft Matter.

[100] Gazit E. (2007). Self-Assembled Peptide Nanostructures: The Design of Molecular Building Blocks and Their Technological Utilization. Chem. Soc. Rev..

[101] Habibi N., Soumetz F.C., Giulianelli M., Pastorino L., Herrera O., Sbrana F., Raiteri R., Ruggiero C. Self-Assembly and Recrystallization of Bacterial S-Layer Proteins of *Bacillus Sphaericus* and *Bacillus Thuringiensis* on Silicone, Mica and Quartz Crystal Supports. Proceedings of the 2010 Annual International Conference of the IEEE Engineering in Medicine and Biology.

[102] Detzel C.J., Larkin A.L., Rajagopalan P. (2011). Polyelectrolyte Multilayers in Tissue Engineering. Tissue Eng. Part B Rev..

[103] Habibi N., Kamaly N., Memic A., Shafiee H. (2016). Self-Assembled Peptide-Based Nanostructures: Smart Nanomaterials toward Targeted Drug Delivery. Nano Today.

[104] Mandal D., Nasrolahi Shirazi A., Parang K. (2014). Self-Assembly of Peptides to Nanostructures. Org. Biomol. Chem..

[105] Smart S.K., Cassady A.I., Lu G.Q., Martin D.J. (2006). The Biocompatibility of Carbon Nanotubes. Carbon.

[106] Reches M., Gazit E. (2006). Designed Aromatic Homo-Dipeptides: Formation of Ordered Nanostructures and Potential Nanotechnological Applications. Phys. Biol..

[107] Silva R.F., Araújo D.R., Silva E.R., Ando R.A., Alves W.A. (2013). L-Diphenylalanine Microtubes As a Potential Drug-Delivery System: Characterization, Release Kinetics, and Cytotoxicity. Langmuir.

[108] Xie J., Lee S., Chen X. (2010). Nanoparticle-Based Theranostic Agents. Adv. Drug Deliv. Rev..

[109] Kamaly N., Kalber T., Thanou M., Bell J.D., Miller A.D. (2009). Folate Receptor Targeted Bimodal Liposomes for Tumor Magnetic Resonance Imaging. Bioconjug. Chem..

[110] Liu W., Nie L., Li F., Aguilar Z.P., Xu H., Xiong Y., Fu F., Xu H. (2016). Folic Acid Conjugated Magnetic Iron Oxide Nanoparticles for Nondestructive Separation and Detection of Ovarian Cancer Cells from Whole Blood. Biomater. Sci..

[111] Kim I.-B., Shin H., Garcia A.J., Bunz U.H.F. (2007). Use of a Folate−PPE Conjugate To Image Cancer Cells in vitro. Bioconjug. Chem..

[112] Sega E.I., Low P.S. (2008). Tumor Detection Using Folate Receptor-Targeted Imaging Agents. Cancer Metastasis Rev..

[113] Sun C., Lee J.S.H., Zhang M. (2008). Magnetic Nanoparticles in MR Imaging and Drug Delivery. Adv. Drug Deliv. Rev..

[114] Tegafaw T., Liu S., Ahmad M.Y., Saidi A.K., Zhao D., Liu Y., Nam S.-W., Chang Y., Lee G.H. (2023). Magnetic Nanoparticle-Based High-Performance Positive and Negative Magnetic Resonance Imaging Contrast Agents. Pharmaceutics.

[115] Jiang Q.L., Zheng S.W., Hong R.Y., Deng S.M., Guo L., Hu R.L., Gao B., Huang M., Cheng L.F., Liu G.H. (2014). Folic Acid-Conjugated Fe3O4 Magnetic Nanoparticles for Hyperthermia and MRI in vitro and in vivo. Appl. Surf. Sci..

[116] Honarmand D., Ghoreishi S.M., Habibi N., Nicknejad E.T. (2016). Controlled Release of Protein from Magnetite–Chitosan Nanoparticles Exposed to an Alternating Magnetic Field. J. Appl. Polym. Sci..

[117] Castillo J.J., Rindzevicius T., Novoa L.V., Svendsen W.E., Rozlosnik N., Boisen A., Escobar P., Martínez F., Castillo-León J. (2013). Non-Covalent Conjugates of Single-Walled Carbon Nanotubes and Folic Acid for Interaction with Cells over-Expressing Folate Receptors. J. Mater. Chem. B.

[118] Zhang Z., Jia J., Lai Y., Ma Y., Weng J., Sun L. (2010). Conjugating Folic Acid to Gold Nanoparticles through Glutathione for Targeting and Detecting Cancer Cells. Bioorg. Med. Chem..

[119] Lee R.J., Low P.S. (1994). Delivery of Liposomes into Cultured KB Cells via Folate Receptor-Mediated Endocytosis. J. Biol. Chem..

[120] Najahi-Missaoui W., Arnold R.D., Cummings B.S. (2021). Safe Nanoparticles: Are We There Yet?. Int. J. Mol. Sci..

[121] Huang Y., Hsu J.C., Koo H., Cormode D.P. (2022). Repurposing Ferumoxytol: Diagnostic and Therapeutic Applications of an FDA-Approved Nanoparticle. Theranostics.

[122] Emtiazi G., Zohrabi T., Lee L.Y., Habibi N., Zarrabi A. (2017). Covalent Diphenylalanine Peptide Nanotube Conjugated to Folic Acid/Magnetic Nanoparticles for Anti-Cancer Drug Delivery. J. Drug Deliv. Sci. Technol..

[123] Bukowski K., Kciuk M., Kontek R. (2020). Mechanisms of Multidrug Resistance in Cancer Chemotherapy. Int. J. Mol. Sci..

[124] Kartal-Yandim M., Adan-Gokbulut A., Baran Y. (2016). Molecular Mechanisms of Drug Resistance and Its Reversal in Cancer. Crit. Rev. Biotechnol..

[125] Resnier P., Montier T., Mathieu V., Benoit J.-P., Passirani C. (2013). A Review of the Current Status of SiRNA Nanomedicines in the Treatment of Cancer. Biomaterials.

[126] Kanasty R., Dorkin J.R., Vegas A., Anderson D. (2013). Delivery Materials for SiRNA Therapeutics. Nat. Mater..

[127] Mathew A.P., Uthaman S., Cho K.-H., Cho C.-S., Park I.-K. (2018). Injectable Hydrogels for Delivering Biotherapeutic Molecules. Int. J. Biol. Macromol..

[128] Roh Y.H., Ruiz R.C.H., Peng S., Lee J.B., Luo D. (2011). Engineering DNA-Based Functional Materials. Chem. Soc. Rev..

[129] Shao Y., Jia H., Cao T., Liu D. (2017). Supramolecular Hydrogels Based on DNA Self-Assembly. Acc. Chem. Res..

[130] Chen L.-H., Liang N.-W., Huang W.-Y., Liu Y.-C., Ho C.-Y., Kuan C.-H., Huang Y.-F., Wang T.-W. (2023). Supramolecular Hydrogel for Programmable Delivery of Therapeutics to Cancer Multidrug Resistance. Biomater. Adv..

[131] Cavaletti G., Alberti P., Marmiroli P. (2011). Chemotherapy-Induced Peripheral Neurotoxicity in the Era of Pharmacogenomics. Lancet Oncol..

[132] Oun R., Moussa Y.E., Wheate N.J. (2018). The Side Effects of Platinum-Based Chemotherapy Drugs: A Review for Chemists. Dalt. Trans..

[133] Herrmann J. (2020). Vascular Toxic Effects of Cancer Therapies. Nat. Rev. Cardiol..

[134] Larsson M., Huang W.-T., Liu D.-M., Losic D. (2017). Local Co-Administration of Gene-Silencing RNA and Drugs in Cancer Therapy: State-of-the Art and Therapeutic Potential. Cancer Treat. Rev..

[135] Fu Y., Li X., Ren Z., Mao C., Han G. (2018). Multifunctional Electrospun Nanofibers for Enhancing Localized Cancer Treatment. Small.

[136] Ji T., Kohane D.S. (2019). Nanoscale Systems for Local Drug Delivery. Nano Today.

[137] Deng H., Dong A., Song J., Chen X. (2019). Injectable Thermosensitive Hydrogel Systems Based on Functional PEG/PCL Block Polymer for Local Drug Delivery. J. Control. Release.

[138] Nance E., Zhang C., Shih T.-Y., Xu Q., Schuster B.S., Hanes J. (2014). Brain-Penetrating Nanoparticles Improve Paclitaxel Efficacy in Malignant Glioma Following Local Administration. ACS Nano.

[139] Tian J., Min Y., Rodgers Z., Au K.M., Hagan C.T., Zhang M., Roche K., Yang F., Wagner K., Wang A.Z. (2017). Co-Delivery of Paclitaxel and Cisplatin with Biocompatible PLGA–PEG Nanoparticles Enhances Chemoradiotherapy in Non-Small Cell Lung Cancer Models. J. Mater. Chem. B.

[140] Yan H., Hou Y.-F., Niu P.-F., Zhang K., Shoji T., Tsuboi Y., Yao F.-Y., Zhao L.-M., Chang J.-B. (2015). Biodegradable PLGA Nanoparticles Loaded with Hydrophobic Drugs: Confocal Raman Microspectroscopic Characterization. J. Mater. Chem. B.

[141] Štaka I., Cadete A., Surikutchi B.T., Abuzaid H., Bradshaw T.D., Alonso M.J., Marlow M. (2019). A Novel Low Molecular Weight Nanocomposite Hydrogel Formulation for Intra-Tumoural Delivery of Anti-Cancer Drugs. Int. J. Pharm..

[142] Unterman S., Charles L.F., Strecker S.E., Kramarenko D., Pivovarchik D., Edelman E.R., Artzi N. (2017). Hydrogel Nanocomposites with Independently Tunable Rheology and Mechanics. ACS Nano.

[143] Jiang L., Ding Y., Xue X., Zhou S., Li C., Zhang X., Jiang X. (2018). Entrapping Multifunctional Dendritic Nanoparticles into a Hydrogel for Local Therapeutic Delivery and Synergetic Immunochemotherapy. Nano Res..

[144] Xu X., Huang Z., Huang Z., Zhang X., He S., Sun X., Shen Y., Yan M., Zhao C. (2017). Injectable, NIR/PH-Responsive Nanocomposite Hydrogel as Long-Acting Implant for Chemophotothermal Synergistic Cancer Therapy. ACS Appl. Mater. Interfaces.

[145] Wang W., Song H., Zhang J., Li P., Li C., Wang C., Kong D., Zhao Q. (2015). An Injectable, Thermosensitive and Multicompartment Hydrogel for Simultaneous Encapsulation and Independent Release of a Drug Cocktail as an Effective Combination Therapy Platform. J. Control. Release.

[146] Dong X., Yang A., Bai Y., Kong D., Lv F. (2020). Dual Fluorescence Imaging-Guided Programmed Delivery of Doxorubicin and CpG Nanoparticles to Modulate Tumor Microenvironment for Effective Chemo-Immunotherapy. Biomaterials.

[147] Miki T., Mizutani Y., Nonomura N., Nomoto T., Nakao M., Saiki S., Kotake T., Okuyama A. (2002). Irinotecan plus Cisplatin Has Substantial Antitumor Effect as Salvage Chemotherapy against Germ Cell Tumors. Cancer Interdiscip. Int. J. Am. Cancer Soc..

[148] Morris P.G., Oda J., Heinemann M.-H., Ilson D.H. (2010). Choroidal Metastases From Esophageal Adenocarcinoma Responding to Chemotherapy With Cisplatin and Irinotecan. J. Clin. Oncol..

[149] Lee Y., Han J.-Y., Moon S.H., Nam B.-H., Lim K.Y., Lee G.K., Kim H.T., Yun T., An H.J., Lee J.S. (2017). Incorporating Erlotinib or Irinotecan Plus Cisplatin into Chemoradiotherapy for Stage III Non-Small Cell Lung Cancer According to EGFR Mutation Status. Cancer Res. Treat..

[150] Wu C., Wang C., Sun L., Xu K., Zhong W. (2020). PLGA Nanoparticle-Reinforced Supramolecular Peptide Hydrogels for Local Delivery of Multiple Drugs with Enhanced Synergism. Soft Matter.

[151] Douillard J.-Y., Sobrero A., Carnaghi C., Comella P., Díaz-Rubio E., Santoro A., Van Cutsem E. (2003). Metastatic Colorectal Cancer: Integrating Irinotecan into Combination and Sequential Chemotherapy. Ann. Oncol..

[152] Simkens L.H.J., van Tinteren H., May A., ten Tije A.J., Creemers G.-J.M., Loosveld O.J.L., de Jongh F.E., Erdkamp F.L.G., Erjavec Z., van der Torren A.M.E. (2015). Maintenance Treatment with Capecitabine and Bevacizumab in Metastatic Colorectal Cancer (CAIRO3): A Phase 3 Randomised Controlled Trial of the Dutch Colorectal Cancer Group. Lancet.

[153] Ducreux M., Malka D., Mendiboure J., Etienne P.-L., Texereau P., Auby D., Rougier P., Gasmi M., Castaing M., Abbas M. (2011). Sequential versus Combination Chemotherapy for the Treatment of Advanced Colorectal Cancer (FFCD 2000–05): An Open-Label, Randomised, Phase 3 Trial. Lancet Oncol..

[154] Cardoso F., Bedard P.L., Winer E.P., Pagani O., Senkus-Konefka E., Fallowfield L.J., Kyriakides S., Costa A., Cufer T., Albain K.S. (2009). International Guidelines for Management of Metastatic Breast Cancer: Combination vs Sequential Single-Agent Chemotherapy. JNCI J. Natl. Cancer Inst..

[155] Wang Y., Zhao Q., Han N., Bai L., Li J., Liu J., Che E., Hu L., Zhang Q., Jiang T. (2015). Mesoporous Silica Nanoparticles in Drug Delivery and Biomedical Applications. Nanomed. Nanotechnol. Biol. Med..

[156] Kiew S.F., Kiew L.V., Lee H.B., Imae T., Chung L.Y. (2016). Assessing Biocompatibility of Graphene Oxide-Based Nanocarriers: A Review. J. Control. Release.

[157] Parveen S., Misra R., Sahoo S.K. (2012). Nanoparticles: A Boon to Drug Delivery, Therapeutics, Diagnostics and Imaging. Nanomed. Nanotechnol. Biol. Med..

[158] Masood F. (2016). Polymeric Nanoparticles for Targeted Drug Delivery System for Cancer Therapy. Mater. Sci. Eng. C.

[159] Li J., Mooney D.J. (2016). Designing Hydrogels for Controlled Drug Delivery. Nat. Rev. Mater..

[160] Dimatteo R., Darling N.J., Segura T. (2018). In situ Forming Injectable Hydrogels for Drug Delivery and Wound Repair. Adv. Drug Deliv. Rev..

[161] Fakhari A., Anand Subramony J. (2015). Engineered In-Situ Depot-Forming Hydrogels for Intratumoral Drug Delivery. J. Control. Release.

[162] Shen B., Lu D., Zhai W., Zheng W. (2013). Synthesis of Graphene by Low-Temperature Exfoliation and Reduction of Graphite Oxide under Ambient Atmosphere. J. Mater. Chem. C.

[163] Deb A., Vimala R. (2018). Camptothecin Loaded Graphene Oxide Nanoparticle Functionalized with Polyethylene Glycol and Folic Acid for Anticancer Drug Delivery. J. Drug Deliv. Sci. Technol..

[164] Deb A., Andrews N.G., Raghavan V. (2018). Natural Polymer Functionalized Graphene Oxide for Co-Delivery of Anticancer Drugs: In-Vitro and in-Vivo. Int. J. Biol. Macromol..

[165] Tran T.H., Nguyen H.T., Pham T.T., Choi J.Y., Choi H.-G., Yong C.S., Kim J.O. (2015). Development of a Graphene Oxide Nanocarrier for Dual-Drug Chemo-Phototherapy to Overcome Drug Resistance in Cancer. ACS Appl. Mater. Interfaces.

[166] Huang Y.-S., Lu Y.-J., Chen J.-P. (2017). Magnetic Graphene Oxide as a Carrier for Targeted Delivery of Chemotherapy Drugs in Cancer Therapy. J. Magn. Magn. Mater..

[167] Hashemi M., Yadegari A., Yazdanpanah G., Jabbehdari S., Omidi M., Tayebi L. (2016). Functionalized R9–Reduced Graphene Oxide as an Efficient Nano-Carrier for Hydrophobic Drug Delivery. RSC Adv..

[168] Xu Z., Zhu S., Wang M., Li Y., Shi P., Huang X. (2015). Delivery of Paclitaxel Using PEGylated Graphene Oxide as a Nanocarrier. ACS Appl. Mater. Interfaces.

[169] Lv Y., Tao L., Annie Bligh S.W., Yang H., Pan Q., Zhu L. (2016). Targeted Delivery and Controlled Release of Doxorubicin into Cancer Cells Using a Multifunctional Graphene Oxide. Mater. Sci. Eng. C.

[170] Yang K., Feng L., Liu Z. (2016). Stimuli Responsive Drug Delivery Systems Based on Nano-Graphene for Cancer Therapy. Adv. Drug Deliv. Rev..

[171] Zhang Q., Wu Z., Li N., Pu Y., Wang B., Zhang T., Tao J. (2017). Advanced Review of Graphene-Based Nanomaterials in Drug Delivery Systems: Synthesis, Modification, Toxicity and Application. Mater. Sci. Eng. C.

[172] Dexter A.F., Fletcher N.L., Creasey R.G., Filardo F., Boehm M.W., Jack K.S. (2017). Fabrication and Characterization of Hydrogels Formed from Designer Coiled-Coil Fibril-Forming Peptides. RSC Adv..

[173] Haines-Butterick L., Rajagopal K., Branco M., Salick D., Rughani R., Pilarz M., Lamm M.S., Pochan D.J., Schneider J.P. (2007). Controlling Hydrogelation Kinetics by Peptide Design for Three-Dimensional Encapsulation and Injectable Delivery of Cells. Proc. Natl. Acad. Sci. USA.

[174] Branco M.C., Pochan D.J., Wagner N.J., Schneider J.P. (2009). Macromolecular Diffusion and Release from Self-Assembled β-Hairpin Peptide Hydrogels. Biomaterials.

[175] Vashist A., Kaushik A., Ghosal A., Bala J., Nikkhah-Moshaie R., Wani W.A., Manickam P., Nair M. (2018). Nanocomposite Hydrogels: Advances in Nanofillers Used for Nanomedicine. Gels.

[176] Liu M., Huang P., Wang W., Feng Z., Zhang J., Deng L., Dong A. (2019). An Injectable Nanocomposite Hydrogel Co-Constructed with Gold Nanorods and Paclitaxel-Loaded Nanoparticles for Local Chemo-Photothermal Synergetic Cancer Therapy. J. Mater. Chem. B.

[177] Basso J., Miranda A., Nunes S., Cova T., Sousa J., Vitorino C., Pais A. (2018). Hydrogel-Based Drug Delivery Nanosystems for the Treatment of Brain Tumors. Gels.

[178] Constantin M., Bucatariu S.-M., Doroftei F., Fundueanu G. (2017). Smart Composite Materials Based on Chitosan Microspheres Embedded in Thermosensitive Hydrogel for Controlled Delivery of Drugs. Carbohydr. Polym..

[179] Lammers T., Subr V., Ulbrich K., Peschke P., Huber P.E., Hennink W.E., Storm G. (2009). Simultaneous Delivery of Doxorubicin and Gemcitabine to Tumors in vivo Using Prototypic Polymeric Drug Carriers. Biomaterials.

[180] Wang C., Zhang G., Liu G., Hu J., Liu S. (2017). Photo- and Thermo-Responsive Multicompartment Hydrogels for Synergistic Delivery of Gemcitabine and Doxorubicin. J. Control. Release.

[181] Liu D., Chen Y., Feng X., Deng M., Xie G., Wang J., Zhang L., Liu Q., Yuan P. (2014). Micellar Nanoparticles Loaded with Gemcitabine and Doxorubicin Showed Synergistic Effect. Colloids Surf. B Biointerfaces.

[182] Vogus D.R., Evans M.A., Pusuluri A., Barajas A., Zhang M., Krishnan V., Nowak M., Menegatti S., Helgeson M.E., Squires T.M. (2017). A Hyaluronic Acid Conjugate Engineered to Synergistically and Sequentially Deliver Gemcitabine and Doxorubicin to Treat Triple Negative Breast Cancer. J. Control. Release.

[183] Vogus D.R., Pusuluri A., Chen R., Mitragotri S. (2018). Schedule Dependent Synergy of Gemcitabine and Doxorubicin: Improvement of in vitro Efficacy and Lack of in vitro-in vivo Correlation. Bioeng. Transl. Med..

[184] Schneible J.D., Shi K., Young A.T., Ramesh S., He N., Dowdey C.E., Dubnansky J.M., Lilova R.L., Gao W., Santiso E. (2020). Modified Gaphene Oxide (GO) Particles in Peptide Hydrogels: A Hybrid System Enabling Scheduled Delivery of Synergistic Combinations of Chemotherapeutics. J. Mater. Chem. B.

[185] Salmaso S., Caliceti P. (2013). Stealth Properties to Improve Therapeutic Efficacy of Drug Nanocarriers. J. Drug Deliv..

[186] Nel A., Ruoslahti E., Meng H. (2017). New Insights into “Permeability” as in the Enhanced Permeability and Retention Effect of Cancer Nanotherapeutics. ACS Nano.

[187] Ravi Kiran A.V.V.V., Kusuma Kumari G., Krishnamurthy P.T., Khaydarov R.R. (2021). Tumor Microenvironment and Nanotherapeutics: Intruding the Tumor Fort. Biomater. Sci..

[188] Donahue N.D., Acar H., Wilhelm S. (2019). Concepts of Nanoparticle Cellular Uptake, Intracellular Trafficking, and Kinetics in Nanomedicine. Adv. Drug Deliv. Rev..

[189] Sabourian P., Yazdani G., Ashraf S.S., Frounchi M., Mashayekhan S., Kiani S., Kakkar A. (2020). Effect of Physico-Chemical Properties of Nanoparticles on Their Intracellular Uptake. Int. J. Mol. Sci..

[190] Mukai H., Ogawa K., Kato N., Kawakami S. (2022). Recent Advances in Lipid Nanoparticles for Delivery of Nucleic Acid, MRNA, and Gene Editing-Based Therapeutics. Drug Metab. Pharmacokinet..

[191] Zeb A., Gul M., Nguyen T.-T.-L., Maeng H.-J. (2022). Controlled Release and Targeted Drug Delivery with Poly(Lactic-Co-Glycolic Acid) Nanoparticles: Reviewing Two Decades of Research. J. Pharm. Investig..

[192] Gallo E., Rosa E., Diaferia C., Rossi F., Tesauro D., Accardo A. (2020). Systematic Overview of Soft Materials as a Novel Frontier for MRI Contrast Agents. RSC Adv..

[193] Yee Kuen C., Masarudin M.J. (2022). Chitosan Nanoparticle-Based System: A New Insight into the Promising Controlled Release System for Lung Cancer Treatment. Molecules.

[194] Sivaram A.J., Rajitha P., Maya S., Jayakumar R., Sabitha M. (2015). Nanogels for Delivery, Imaging and Therapy. WIREs Nanomed. Nanobiotechnol..

[195] Nik M.E., Malaekeh-Nikouei B., Amin M., Hatamipour M., Teymouri M., Sadeghnia H.R., Iranshahi M., Jaafari M.R. (2019). Liposomal Formulation of Galbanic Acid Improved Therapeutic Efficacy of Pegylated Liposomal Doxorubicin in Mouse Colon Carcinoma. Sci. Rep..

[196] Joniec A., Sek S., Krysinski P. (2016). Magnetoliposomes as Potential Carriers of Doxorubicin to Tumours. Chem.-A Eur. J..

[197] Haghiralsadat F., Amoabediny G., Sheikhha M.H., Zandieh-doulabi B., Naderinezhad S., Helder M.N., Forouzanfar T. (2017). New Liposomal Doxorubicin Nanoformulation for Osteosarcoma: Drug Release Kinetic Study Based on Thermo and PH Sensitivity. Chem. Biol. Drug Des..

[198] Gavas S., Quazi S., Karpiński T.M. (2021). Nanoparticles for Cancer Therapy: Current Progress and Challenges. Nanoscale Res. Lett..

[199] Smaldone G., Rosa E., Gallo E., Diaferia C., Morelli G., Stornaiuolo M., Accardo A. (2023). Caveolin-Mediated Internalization of Fmoc-FF Nanogels in Breast Cancer Cell Lines. Pharmaceutics.

[200] Carniato F., Tei L., Botta M., Ravera E., Fragai M., Parigi G., Luchinat C. (2020). 1H NMR Relaxometric Study of Chitosan-Based Nanogels Containing Mono- and Bis-Hydrated Gd(III) Chelates: Clues for MRI Probes of Improved Sensitivity. ACS Appl. Bio Mater..

[201] Daly A.C., Riley L., Segura T., Burdick J.A. (2020). Hydrogel Microparticles for Biomedical Applications. Nat. Rev. Mater..

[202] Rosa E., Diaferia C., Gallo E., Morelli G., Accardo A. (2020). Stable Formulations of Peptide-Based Nanogels. Molecules.

[203] Diaferia C., Rosa E., Morelli G., Accardo A. (2022). Fmoc-Diphenylalanine Hydrogels: Optimization of Preparation Methods and Structural Insights. Pharmaceuticals.

[204] Diaferia C., Rosa E., Accardo A., Morelli G. (2022). Peptide-Based Hydrogels as Delivery Systems for Doxorubicin. J. Pept. Sci..

[205] Gallo E., Diaferia C., Rosa E., Smaldone G., Morelli G., Accardo A. (2021). Peptide-Based Hydrogels and Nanogels for Delivery of Doxorubicin. Int. J. Nanomed..

[206] Hegde P.S., Karanikas V., Evers S. (2016). The Where, the When, and the How of Immune Monitoring for Cancer Immunotherapies in the Era of Checkpoint Inhibition. Clin. Cancer Res..

[207] Scheper W., Kelderman S., Fanchi L.F., Linnemann C., Bendle G., de Rooij M.A.J., Hirt C., Mezzadra R., Slagter M., Dijkstra K. (2019). Low and Variable Tumor Reactivity of the Intratumoral TCR Repertoire in Human Cancers. Nat. Med..

[208] Galon J., Bruni D. (2019). Approaches to Treat Immune Hot, Altered and Cold Tumours with Combination Immunotherapies. Nat. Rev. Drug Discov..

[209] Hayashi K., Nikolos F., Lee Y.C., Jain A., Tsouko E., Gao H., Kasabyan A., Leung H.E., Osipov A., Jung S.Y. (2020). Tipping the Immunostimulatory and Inhibitory DAMP Balance to Harness Immunogenic Cell Death. Nat. Commun..

[210] Alzeibak R., Mishchenko T.A., Shilyagina N.Y., Balalaeva I.V., Vedunova M.V., Krysko D.V. (2021). Targeting Immunogenic Cancer Cell Death by Photodynamic Therapy: Past, Present and Future. J. Immunother. Cancer.

[211] Fang H., Ang B., Xu X., Huang X., Wu Y., Sun Y., Wang W., Li N., Cao X., Wan T. (2014). TLR4 Is Essential for Dendritic Cell Activation and Anti-Tumor T-Cell Response Enhancement by DAMPs Released from Chemically Stressed Cancer Cells. Cell. Mol. Immunol..

[212] Yu M., Zeng W., Ouyang Y., Liang S., Yi Y., Hao H., Yu J., Liu Y., Nie Y., Wang T. (2022). ATP-Exhausted Nanocomplexes for Intratumoral Metabolic Intervention and Photoimmunotherapy. Biomaterials.

[213] Jin C., Wang Y., Li Y., Li J., Zhou S., Yu J., Wang Z., Yu Y., Zhang H., Wang D. (2021). Doxorubicin-Near Infrared Dye Conjugate Induces Immunogenic Cell Death to Enhance Cancer Immunotherapy. Int. J. Pharm..

[214] Jiang M., Zeng J., Zhao L., Zhang M., Ma J., Guan X., Zhang W. (2021). Chemotherapeutic Drug-Induced Immunogenic Cell Death for Nanomedicine-Based Cancer Chemo–Immunotherapy. Nanoscale.

[215] Zheng P., Ding B., Jiang Z., Xu W., Li G., Ding J., Chen X. (2021). Ultrasound-Augmented Mitochondrial Calcium Ion Overload by Calcium Nanomodulator to Induce Immunogenic Cell Death. Nano Lett..

[216] Xia J., Ma S., Zhu X., Chen C., Zhang R., Cao Z., Chen X., Zhang L., Zhu Y., Zhang S. (2023). Versatile Ginsenoside Rg3 Liposomes Inhibit Tumor Metastasis by Capturing Circulating Tumor Cells and Destroying Metastatic Niches. Sci. Adv..

[217] Chen M., Qiao Y., Cao J., Ta L., Ci T., Ke X. (2022). Biomimetic Doxorubicin/Ginsenoside Co-Loading Nanosystem for Chemoimmunotherapy of Acute Myeloid Leukemia. J. Nanobiotechnol..

[218] Yaroslavov A.A., Efimova A.A., Krasnikov E.A., Trosheva K.S., Popov A.S., Melik-Nubarov N.S., Krivtsov G.G. (2021). Chitosan-Based Multi-Liposomal Complexes: Synthesis, Biodegradability and Cytotoxicity. Int. J. Biol. Macromol..

[219] Li J., Fu J., Tian X., Hua T., Poon T., Koo M., Chan W. (2022). Characteristics of Chitosan Fiber and Their Effects towards Improvement of Antibacterial Activity. Carbohydr. Polym..

[220] Hu Y., Du Y., Liu N., Liu X., Meng T., Cheng B., He J., You J., Yuan H., Hu F. (2015). Selective Redox-Responsive Drug Release in Tumor Cells Mediated by Chitosan Based Glycolipid-like Nanocarrier. J. Control. Release.

[221] Yang H., Liu T., Xu Y., Su G., Liu T., Yu Y., Xu B. (2021). Protein Corona Precoating on Redox-Responsive Chitosan-Based Nano-Carriers for Improving the Therapeutic Effect of Nucleic Acid Drugs. Carbohydr. Polym..

[222] Wang Y., Qian J., Yang M., Xu W., Wang J., Hou G., Ji L., Suo A. (2019). Doxorubicin/Cisplatin Co-Loaded Hyaluronic Acid/Chitosan-Based Nanoparticles for in vitro Synergistic Combination Chemotherapy of Breast Cancer. Carbohydr. Polym..

[223] Zhang P., Qin C., Liu N., Zhou X., Chu X., Lv F., Gu Y., Yin L., Liu J., Zhou J. (2022). The Programmed Site-Specific Delivery of LY3200882 and PD-L1 SiRNA Boosts Immunotherapy for Triple-Negative Breast Cancer by Remodeling Tumor Microenvironment. Biomaterials.

[224] Jin X., Fu Q., Gu Z., Zhang Z., Lv H. (2020). Injectable Corilagin/Low Molecular Weight Chitosan/PLGA-PEG-PLGA Thermosensitive Hydrogels for Localized Cancer Therapy and Promoting Drug Infiltration by Modulation of Tumor Microenvironment. Int. J. Pharm..

[225] Yu Y., Zu C., He D., Li Y., Chen Q., Chen Q., Wang H., Wang R., Chaurasiya B., Zaro J.L. (2021). PH-Dependent Reversibly Activatable Cell-Penetrating Peptides Improve the Antitumor Effect of Artemisinin-Loaded Liposomes. J. Colloid Interface Sci..

[226] Xie J., Bi Y., Zhang H., Dong S., Teng L., Lee R.J., Yang Z. (2020). Cell-Penetrating Peptides in Diagnosis and Treatment of Human Diseases: From Preclinical Research to Clinical Application. Front. Pharmacol..

[227] Copolovici D.M., Langel K., Eriste E., Langel Ü. (2014). Cell-Penetrating Peptides: Design, Synthesis, and Applications. ACS Nano.

[228] Kishore Deb P., Al-Attraqchi O., Chandrasekaran B., Paradkar A., Tekade R.K. (2019). Protein/Peptide Drug Delivery Systems: Practical Considerations in Pharmaceutical Product Development. Basic Fundam. Drug Deliv..

[229] Wu H., Wei G., Luo L., Li L., Gao Y., Tan X., Wang S., Chang H., Liu Y., Wei Y. (2022). Ginsenoside Rg3 Nanoparticles with Permeation Enhancing Based Chitosan Derivatives Were Encapsulated with Doxorubicin by Thermosensitive Hydrogel and Anti-Cancer Evaluation of Peritumoral Hydrogel Injection Combined with PD-L1 Antibody. Biomater. Res..

[230] Mirzavi F., Barati M., Vakili-Ghartavol R., Roshan M.K., Mashreghi M., Soukhtanloo M., Jaafari M.R. (2022). Pegylated Liposomal Encapsulation Improves the Antitumor Efficacy of Combretastatin A4 in Murine 4T1 Triple-Negative Breast Cancer Model. Int. J. Pharm..

[231] Guilbaud-Chéreau C., Dinesh B., Schurhammer R., Collin D., Bianco A., Ménard-Moyon C. (2019). Protected Amino Acid-Based Hydrogels Incorporating Carbon Nanomaterials for Near-Infrared Irradiation-Triggered Drug Release. Appl. Mater. Interfaces.

[232] Biswas S., Rasale D.B., Das A.K. (2016). Blue Light Emitting Self-Healable Graphene Quantum Dots Embedded Hydrogels. RSC Adv..

[233] Wu J., Chen A., Qin M., Huang R., Zhang G., Xue B., Wei J., Li Y., Cao Y., Wang W. (2015). Hierarchical Construction of a Mechanically Stable Peptide–Graphene Oxide Hybrid Hydrogel for Drug Delivery and Pulsatile Triggered Release in vivo. Nanoscale.

[234] Li B., Criado-Gonzalez M., Adam A., Bizeau J., Mélart C., Carvalho A., Bégin S., Bégin D., Jierry L., Mertz D. (2022). Peptide Hydrogels Assembled from Enzyme-Adsorbed Mesoporous Silica Nanostructures for Thermoresponsive Doxorubicin Release. ACS Appl. Nano Mater..

[235] Criado-Gonzalez M., Fores J.R., Carvalho A., Blanck C., Schmutz M., Kocgozlu L., Schaaf P., Jierry L., Boulmedais F. (2019). Phase Separation in Supramolecular Hydrogels Based on Peptide Self-Assembly from Enzyme-Coated Nanoparticles. Langmuir.

[236] Ligorio C., Zhou M., Wychowaniec J.K., Zhu X., Bartlam C., Miller A.F., Vijayaraghavan A., Hoyland J.A., Saiani A. (2019). Graphene Oxide Containing Self-Assembling Peptide Hybrid Hydrogels as a Potential 3D Injectable Cell Delivery Platform for Intervertebral Disc Repair Applications. Acta Biomater..

[237] Li K., Zhang Z., Li D., Zhang W., Yu X., Liu W., Gong C., Wei G., Su Z. (2018). Biomimetic Ultralight, Highly Porous, Shape-Adjustable, and Biocompatible 3D Graphene Minerals via Incorporation of Self-Assembled Peptide Nanosheets. Adv. Funct. Mater..

[238] Su Z., Shen H., Wang H., Wang J., Li J., Nienhaus G.U., Shang L., Wei G. (2015). Motif-Designed Peptide Nanofibers Decorated with Graphene Quantum Dots for Simultaneous Targeting and Imaging of Tumor Cells. Adv. Funct. Mater..

[239] Wen Q., Zhang Y., Li C., Ling S., Yang X., Chen G., Yang Y., Wang Q. (2019). NIR-II Fluorescent Self-Assembled Peptide Nanochain for Ultrasensitive Detection of Peritoneal Metastasis. Angew. Chemie Int. Ed..

